# Chromosome-level genome assembly and population genetic analysis of a near-threatened rosewood species (*Dalbergia cultrata* Pierre Graham ex Benth) provide insights into its evolutionary and cold stress responses

**DOI:** 10.3389/fpls.2023.1212967

**Published:** 2023-09-21

**Authors:** Ping Huang, Changhong Li, Furong Lin, Yu Liu, Yichen Zong, Bin Li, Yongqi Zheng

**Affiliations:** ^1^ State Key Laboratory of Tree Genetics and Breeding, Chinese Academy of Forestry, Beijing, China; ^2^ Laboratory of Forest Silviculture and Tree Cultivation, National Forestry and Grassland Administration, Research Institute of Forestry, Chinese Academy of Forestry, Beijing, China; ^3^ Wenzhou Key Laboratory of Resource Plant Innovation and Utilization, Zhejiang Institute of Subtropical Crops, Zhejiang Academy of Agricultural Sciences, Wenzhou, Zhejiang, China

**Keywords:** Dalbergia cultrata, genome assembly, population evolution, cold stress response, rosewood

## Abstract

*Dalbergia cultrata* Pierre Graham ex Benth (*D. cultrata*) is a precious rosewood tree species that grows in the tropical and subtropical regions of Asia. In this study, we used PacBio long-reading sequencing technology and Hi-C assistance to sequence and assemble the reference genome of *D. cultrata*. We generated 171.47 Gb PacBio long reads and 72.43 Gb Hi-C data and yielded an assembly of 10 pseudochromosomes with a total size of 690.99 Mb and Scaffold N50 of 65.76 Mb. The analysis of specific genes revealed that the triterpenoids represented by lupeol may play an important role in *D. cultrata*’s potential medicinal value. Using the new reference genome, we analyzed the resequencing of 19 *Dalbergia* accessions and found that *D. cultrata* and *D. cochinchinensis* have the latest genetic relationship. Transcriptome sequencing of *D. cultrata* leaves grown under cold stress revealed that MYB transcription factor and E3 ubiquitin ligase may be playing an important role in the cold response of *D. cultrata*. Genome resources and identified genetic variation, especially those genes related to the biosynthesis of phytochemicals and cold stress response, will be helpful for the introduction, domestication, utilization, and further breeding of *Dalbergia* species.

## 1 Introduction

**Figure 1:**
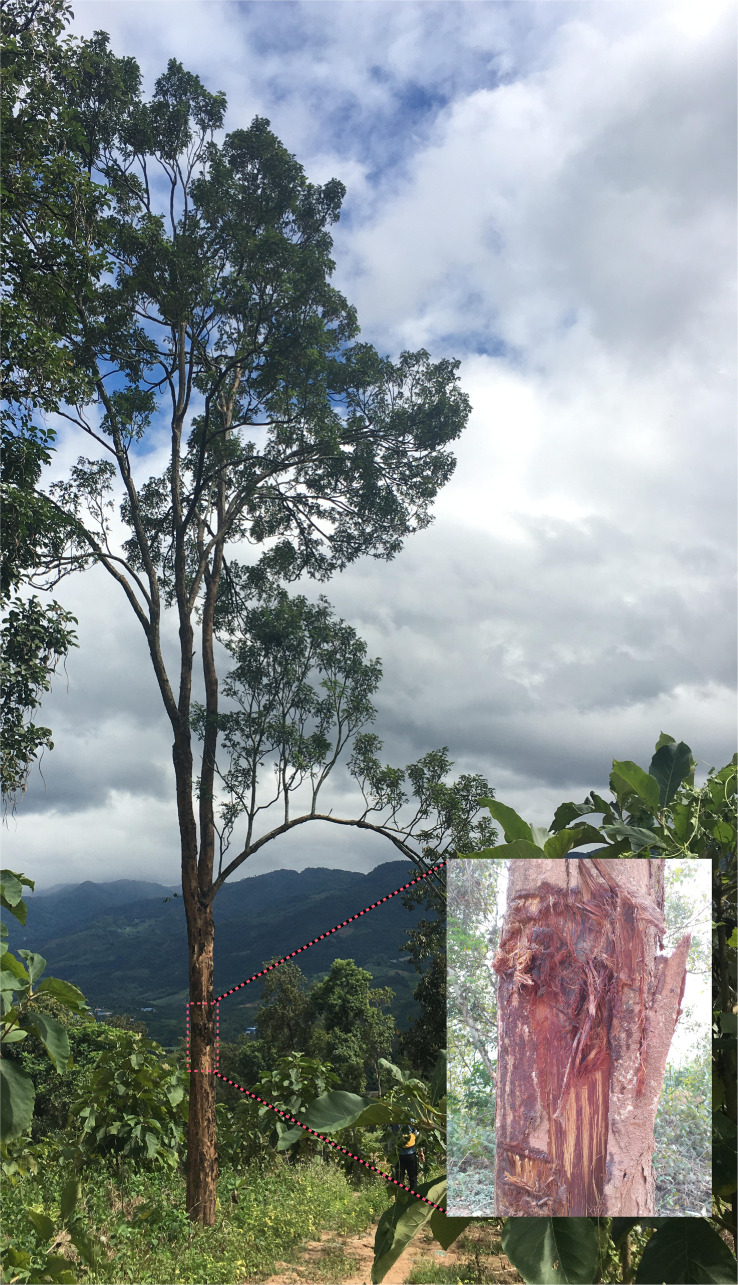
*D.* cultrata plants growing in the wild.

The genus *Dalbergia* belongs to the subfamily Papilionaceae and includes approximately 250 species of trees, shrubs, and woody climbers distributed in tropical and subtropical regions worldwide (Vatanparast et al., 2013). Many highly valuable timber-yielding species in the genus *Dalbergia* are known for their unique dense, durable characteristics, and abundant color variation, and are highly valued in the manufacture of fine musical instruments, arts and crafts, and furniture, including *Dalbergia cultrata* Pierre Graham ex Benth (NCBI: txid862910) (**Figure 1**) and *D. odorifera* T. C. Chen (Song et al., 2019). *D. cultrata* is a deciduous tree species with high ecological and economic value because of the disease, insects, and fire resistance of its valuable rosewood wood (Liu et al., 2019b). Owing to the increasing demand for rosewood around the world, the natural range of *D. cultrata* is now extremely contracted and its status is Near Threatened (NT). It is listed on the Red List of the International Union for Conservation of Nature (IUCN) and on China’s list of wild plants under Class II State protection.

Recent studies on the genus *Dalbergia* have focused on compounds (Sun et al., 2020b; Zhao et al., 2020; Mori-Yasumoto et al., 2021), seed germination (Seng and Cheong, 2020), and potential distribution prediction (Liu et al., 2019b). However, only a few genomic and transcriptomic studies have been conducted on the genus *Dalbergia*, especially *D. cultrata*, and only a few chloroplast genomes (Liu et al., 2019a; Hong et al., 2022; Qin et al., 2022), mitochondrial genomes (Hong et al., 2021), reference transcriptomes (Hung et al., 2020), and the chromosome-level draft genome of *D. odorifera* (Hong et al., 2020) have been reported. The molecular phylogenetic framework of *Dalbergia* genus has been preliminarily established; however, there remains some outstanding issues because of its wide distribution, complex origin, and lack of genetic knowledge. More genomic information will help to solve practical problems in taxonomy and tree breeding.

In this paper, we report a high-quality genome sequence of *D. cultrata* obtained using PacBio sequencing and high-throughput chromosome conformation capture (Hi-C) technology. Detailed information on the *D. cultrata* genome, including repeat sequences, gene annotation, and evolution may help elucidate the biogeography and evolution of genus *Dalbergia* plants and contribute to the understanding of the molecular basis of its resistance to abiotic stress.

## 2 Results

### 2.1 Genome sequencing, assembly, and annotation

To generate a chromosome-leve l genome assembly of *D. cultrata*, Illumina paired-end short read sequencing (~113x), PacBio SMRT sequencing (~248x), and Hi-C sequencing technology (~104x) were used (**Table 1**). The *D. cultrata* genome size was evaluated using *K-mer* method based on Illumina short reads, and the result was ~639.48 Mb, with 47.70% repetitive sequence, 0.78% heterozygosity, and 34.63% GC content (**Figure S1A**). The genome sizes of *D. cultrata* estimated by flow cytometry were 592.3Mb and 643.9Mb using the *Glycine max* and *Oryza sativa* genomes as references (**Figure S1B**), which is similar to the result predicted by the *k-mer* method.

**Table 1:** Sequencing data used for *Dalbergia cultrata* genome assembly and annotation.

Sequencing type	Sequencing platform	Data Bases (Gb)	Data Reads	Coverage (×)
Short reads for genome survey	Illumina NovaSeq 6000	78.11	520,743,140	113
Long reads for contig assembly	PacBio Sequel II	171.47	8,140,676	248
Hi-C reads for chromosome construction	Illumina NovaSeq 6000	72.43	483,835,664	104
Transcriptome long reads of root for genome annotation	Oxford Nanopore Technologies	2.87	2,168,896	–
Transcriptome long reads of branch xylem for genome annotation	Oxford Nanopore Technologies	3	2,404,535	–
Transcriptome long reads of branch phloem for genome annotation	Oxford Nanopore Technologies	2.94	2,305,516	–
Transcriptome long reads of young leaf for genome annotation	Oxford Nanopore Technologies	3.15	2,690,937	–
Transcriptome long reads of leaf for genome annotation	Oxford Nanopore Technologies	3.17	2,529,562	–
Transcriptome long reads of young branch for genome annotation	Oxford Nanopore Technologies	2.82	2,397,084	–

**Figure 2:**
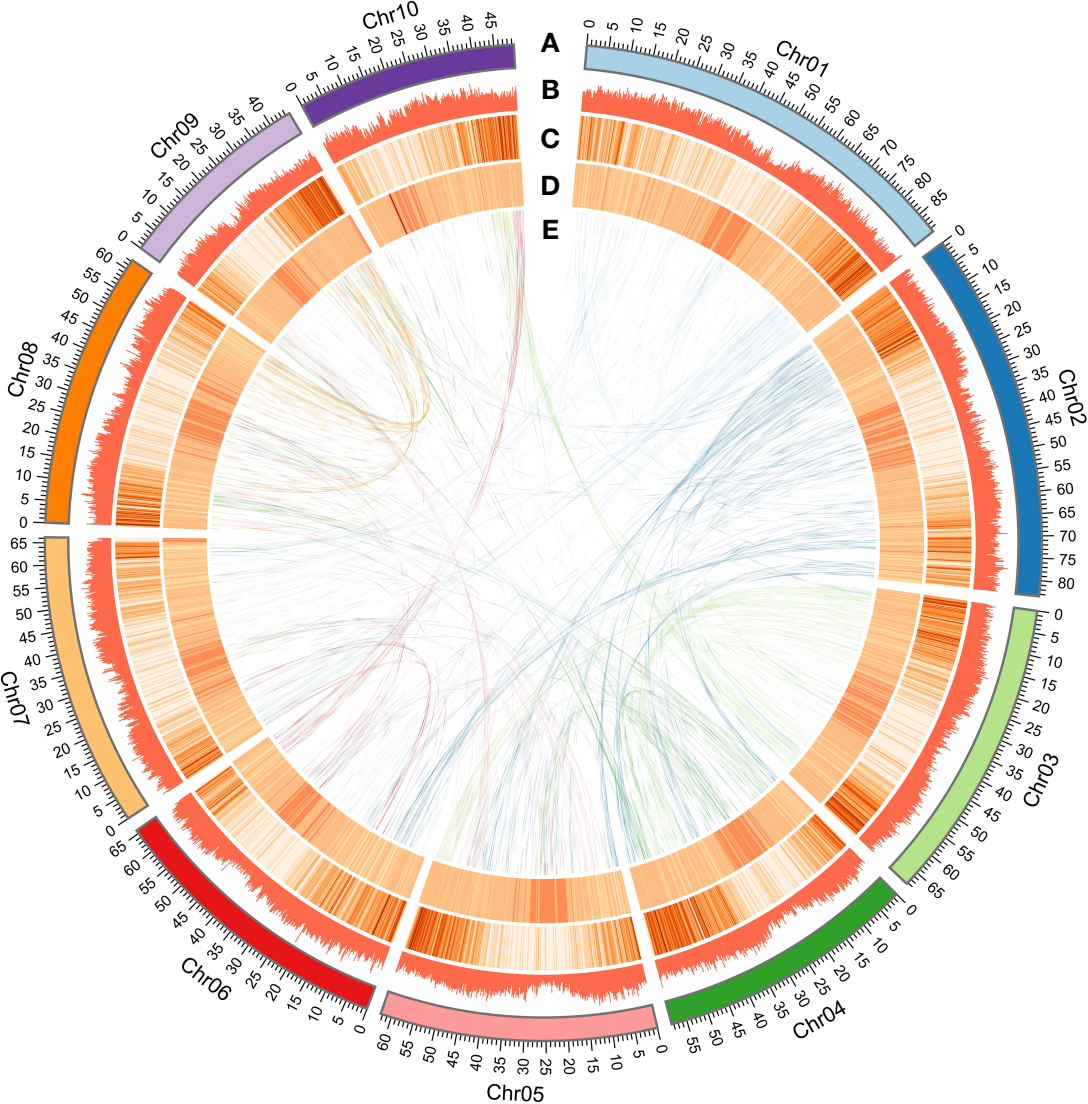
High-quality assembly of 10 chromosomes. **(A)** The high-quality assembly of 10 chromosomes. (**B**) Repeat sequence density (window size 200 kb). **(C)** Gene density (window size of 200 kb). **(D)** GC content density (window size of 200 kb). **(E)** Relationship between syntenic blocks.

A total of 1,083 contigs were assembled and generated a *D. cultrata* genome of 690.90 Mb with a contig N50 of 1.81 Mb and 34.34% GC content using ~171.47Gb Pacbio reads. Furthermore, the 1,083 contigs were clustered into 10 genetic groups based on the Hi-C data, and ~687.26 Mb Hi-C sequence (~99.47%) was anchored onto the 10 pseudochromosomes, of which 95.84% could be oriented (**Figure 2**; **Table 2**). The Contig N50 and Scaffold N50 for the final assembly genome (690.99Mb) after Hi-C error correction were 1.81 Mb and 65.76 Mb, respectively. The heat map of the Hi-C assembly result suggested that the interaction intensity of the diagonal region was stronger than that of the non-diagonal region, indicating that these contigs were well located on the pseudochromosomes (**Figure S2**). Compared to the published *D. odorifera* genome, the newly assembled *D. cultrata* genome has a similar genome size, GC content, and ratio of repeat sequences, while having more coding genes and a longer scaffold N50 (**Table 2**).

**Table 2:** Comparison with *Dalbergia odorifera* genome assemblies and annotated genes.

Assembly feature	*D. odorifera*	*D. cultrata*
Genome size	653.45 Mb	690.99Mb
No. of scaffolds	384	361
Contig N50	5.92 Mb	1.81Mb
Scaffold N50	56.16 Mb	65.76Mb
Longest scaffold	79.61 Mb	89.88Mb
Total number of N	613,549	79,300
Anchored and oriented	94.38	95.84
Repeat region % of genome	52.91	52.7
Predicted gene models	30,310	31,342
Mean coding sequence length	1121.36 bp	4854.156bp
Mean exons per gene	4.93	5.6178
GC content	34.11%	34.34%

Three methods, including Illumina reads alignment, BUSCO evaluation, and whole-genome high long terminal repeat (LTR) assembly index (LAI) score evaluation, were used to assess the assembly integrity of the *D. cultrata* genome. First, more than 99.65% of the Illumina reads were correctly mapped to the final assembled genome (QV value = 33.81). Second, approximately 98.60% and 95.50% of the 1614 highly conserved embryophyte genes in the BUSCO v10 database were identified as complete BUSCOs for the *D. cultrata* genome and annotated protein sequences, respectively (**Table S1**). Moreover, the LAI score of the *D. cultrata* assembly genome was 10.83 (>10), which indicated that the assembly quality of *D. cultrata* was at the reference genome level. Based on these results, the genome assembly quality of *D. cultrata* reached the chromosomal-level reference genome.

Repetitive elements mainly include tandem repeats (TR) and interspersed repeats, among which the second type is transposable elements (TE). In the *D. cultrata* assembled genome, ~52.70% and ~9.03% assembled sequences were annotated as TE and TR, respectively (**Table S2**).

Furthermore, 31,342 protein-coding genes were identified using homology, *ab initio*, and transcriptome predictions. Among them, more than 99% could be annotated using at least one of the following protein-related databases: GO (84.91%), KEGG (78.76%), KOG (58.30%), TrEMBL (99.15%), and NR (99.17%) (**Table S3**), which indicated that the accuracy of gene function prediction was high. Additionally, 267 rRNAs, 605 tRNAs, 120 snRNAs, 122 snoRNAs, and 104 miRNAs were identified.

### 2.2 Evolution of the *Dalbergia cultrata* genome

The phylogenetic tree constructed based on the time of fossil evidence showed that the differentiation time between *D. cultrata* and *D. odorifera* was 5.8–29.81 MYA, between *D. cultrata* and *A. duranensis* was 37.97–52.29 MYA, and between *D. cultrata* and *V. vinifera* was 108.29–135.42 MYA (**Figure S3**).

**Figure 3:**
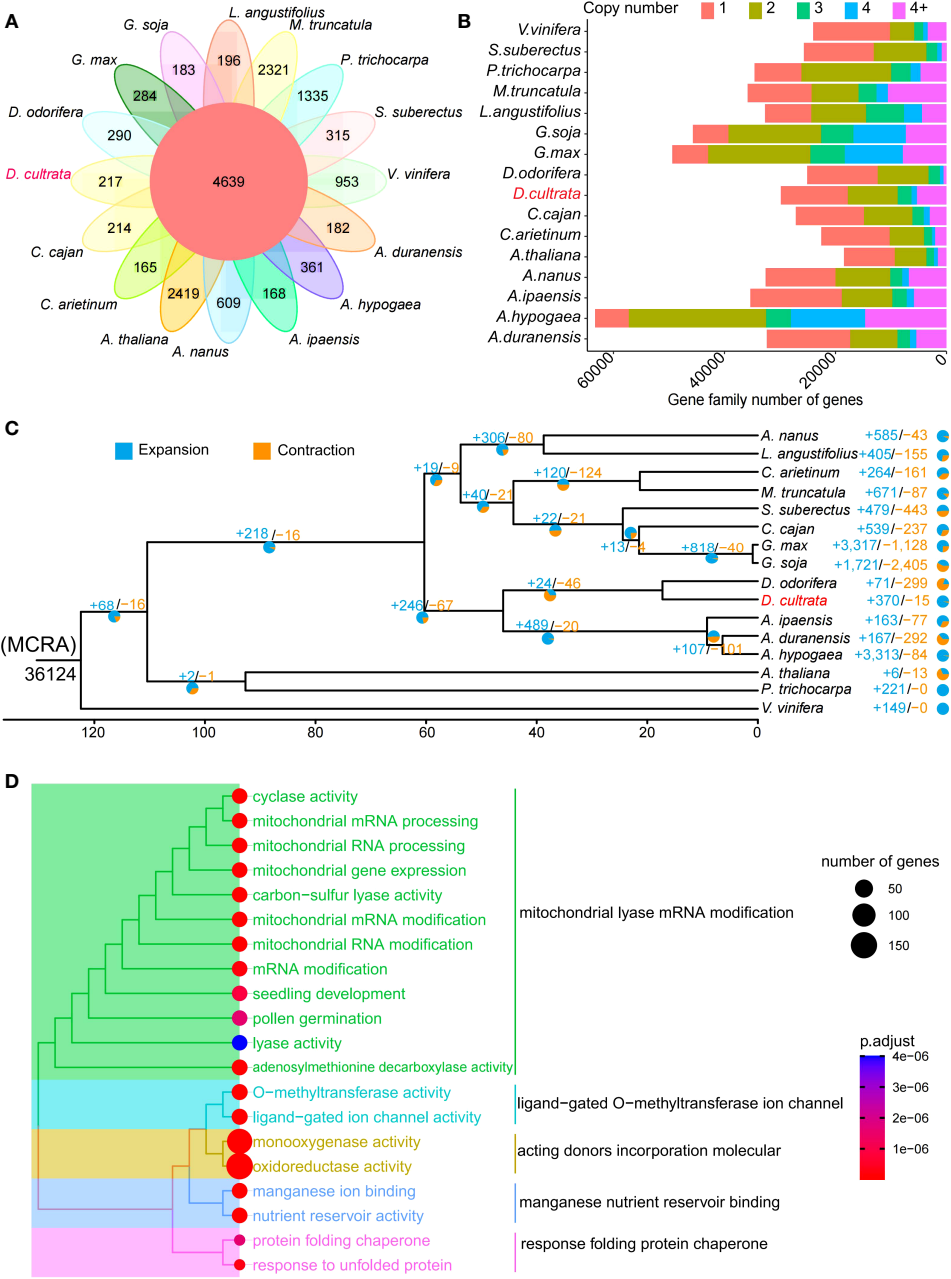
Evolution of the *Dalbergia cultrata* genome. **(A)** A Venn diagram of specific and shared orthologs among 16 species (Vitis vinifera, Cajanus cajan, Cicer arietinum, Populus trichocarpa, Ammopiptanthus nanus, Medicago truncatula, Glycine soja, Arachis hypogaea, Dalbergia odorifera, Dalbergia cultrata, Arachis duranensis, Lupinus angustifolius, Arabidopsis thaliana, Spatholobus suberectus, Arachis ipaensis and Glycine max), identified based on gene family cluster analysis. Each number in the diagram represents the number of gene families within a group. **(B)** The numbers of gene copy in the gene families of the above 16 species. **(C)** Expansion and contraction of gene families. **(D)** GO enrichment analysis of expansion genes (top 20 terms).

There were 4,639 gene families shared by 16 species (**Table S4**), and 217 gene families specific to *D. cultrata* (**Figure 3A**). The copy number of genes within the gene family of *D. cultrata* was mostly one or two (**Figure 3B**). The results of the expansion and contraction of gene families showed that *D. cultrata* had 370 expanded gene families and 15 contracted gene families. However, there were 299 contracted gene families and 71 expanded gene families in *D. odorifera*, which are most closely related to the evolution of *D. cultrata* (**Figure 3C**). GO enrichment analysis of the expanded gene family of *D. cultrata* mainly enriched for mitochondrial lyase mRNA modification, ligand-gated O-methyltransferase ion channel, acting donor incorporation molecule, manganese nutrient reservoir binding, and response folding protein chaperone (**Figure 3D**). A total of 27 positively selected genes were identified, and KEGG enriched four genes and four pathways. *Dcu09G009880* and *Dcu05G025760* were enriched in solute carrier family 8 (sodium/calcium exchanger), *Dcu01G022380* in the chalcone synthase and alpha-mannosidase pathways, and *Dcu03G033380* in the nucleolin pathway (**Table S5**).

Compared with the other 15 genomes, 1,517 specific genes were identified in *D. cultrata*. GO enrichment analysis of these specific genes for aromatic compound bond acting, RNA-directed DNA polymerase activity, GPI anchor biosynthetic process, DNA integration, and cytokinein dehydrogenase activity (**Figure S4A**). KEGG enrichment analysis suggests that these specific genes were mainly enriched for DNA synthase dehydrogenase homogentisate, peroxin−3, phospholipase D1/2, YTH domain-containing family protein, and the AP−3 complex subunit delta pathway (**Figure S4B**; **Table S5**).

### 2.3 Whole-genome duplication analysis

**Figure 4:**
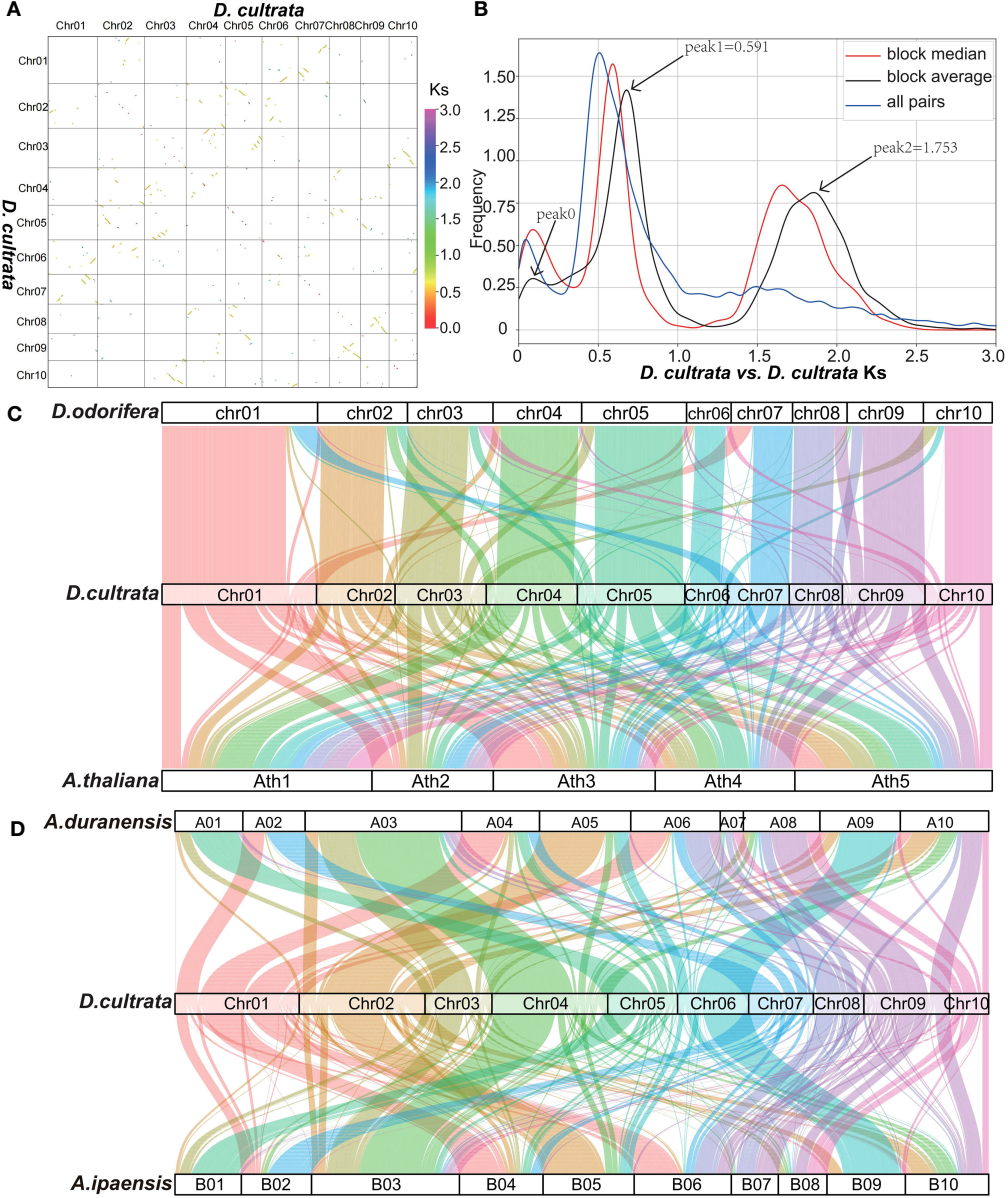
Genome collinearity analysis. **(A)** DNA sequence alignment of the 10 chromosomes of *D. cultrata*. **(B)** *D. cultrata* vs. *D. cultrata* Ks distribution. Combined with a, peak 0 in b was tandem repeat sequence distributed on the diagonal, not a real WGD peak. **(C)** *D. odorifera*, *D. cultrata* and *A. thaliana* gene level collinearity analysis. **(D)** *A. duranensis*, *D. cultrata* and *A.ipaensis* gene level collinearity analysis.

The DNA sequence alignment of the *D. cultrata* genome showed that it experienced two WGD (Whole genome duplication) events, with the young recent one 49.28 MYA (Ks peak1 0.591) and the other 146.1 MYA (Ks peak2 1.753) (**Figures 4A, B**). The results of the genome collinearity analysis showed that *D. cultrata* and *D. odorifera* had good collinearity and indicated that they did not experience new WGD events after separation, whereas *D. cultrata* and *A. thaliana* experienced a new WGD event after separation (**Figure 4C**). The collinearity analysis of *D. cultrata*, *A. duranensis*, and *A. ipaensis* indicated that *D. cultrata* and *Arachis* experienced young recent WGD events in common (**Figure 4D**).

Ks and 4DTv analysis showed that the differentiation time of *D. cultrata* and *D. odorifera* was 6.265 MYA (Ks peak 0.075, 4DTv peak 0.005), that of *D. cultrata* and *A. duranensis* was 41.40 MYA (Ks peak 0.497, 4DTv peak 0.151), and that of *D. cultrata* and *V.vinifera* was 110.3 MYA (Ks peak 1.323, 4DTv peak 0.319) (**Figures S5A**, **B**). The burst time of *D. cultrata* LTR transposon was 0.191 MYA, which was close to 0.185 MYA of *D. odorifera*. The LTR burst time of *A. thaliana* was 0.207 MYA. The LTR burst time was consistent with that of the evolutionary sequence (**Figure S5C**).

The duplication types of genes in the *D. cultrata* genome were divided into five categories, namely WGD (whole-genome duplication), TD (tandem duplication), DSD (dispersed duplication), TRD (transposed duplication), and PD (proximal duplication). Of these, 7,670 gene pairs were of the WGD replication type and 11,108 genes accounting for 40.48% of the five total genes (**Table S6**). The TD and PD types had the highest ratio of Ka/Ks>1, indicating that the main driving forces for recent evolution were tandem and proximal duplications (**Figure S6A**). The smaller and higher peaks in Ks and 4DTv of tandem and proximal duplications also confirmed that these two duplication modes were more active recently. The two main peaks of Ks and 4DTv in WGD also indicated that the *D. cultrata* genome experienced two WGD events (**Figures S6B, C**).

### 2.4 Genome structural variation

The results of the genomic structural variation analysis demonstrated that *D. cultrata* and *D. odorifera* had good collinearity, with inversion and translocation types of structural variation (**Figures S7A, B**). Inversions occurred on all the chromosomes of *D. cultrata* and were the main type of structural variation in the *D. cultrata* genome (**Figure S7B**). A larger inversion occurred in Chr10:43099-9692798 in *D. cultrata*. *D. cultrate*, and *A. duranensis* also showed inversion in this position (**Figure S7C**), *D. odorifera* and *A. duranensis* did not have inversion in this position (**Figure S7D**), indicating that this inversion is an event experienced by *D. cultrata* alone (**Figure S7**).

### 2.5 Genetic variation and population structure

To assess the genetic variation that occurred during evolution and to discover the evolutionary relationships of species of the genus *Dalbergia*, we resequenced the whole genomes of nine accessions and collected 10 accessions from the NCBI, generating a total of 574.4 Gb of reads (**Supplementary Table 1**). We then aligned these reads to the reference genome of *D. cultrata* and identified 89,426,197 high-quality SNPs.

**Figure 5:**
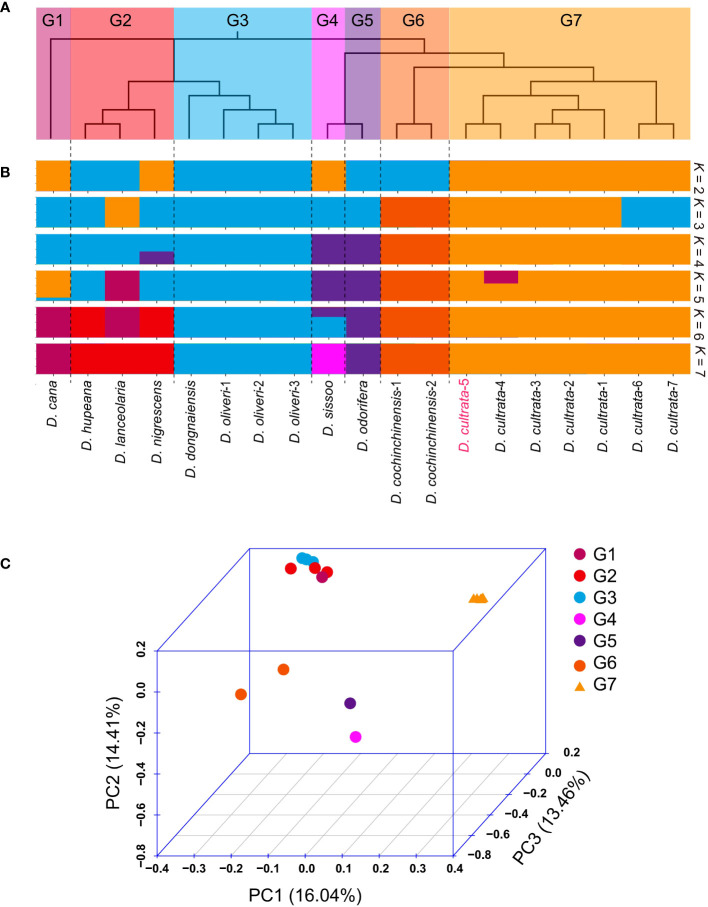
Resequencing and analysis of population structure and evolutionary relationships. **(A)** Phylogenetic tree of 22 resequencing samples. **(B)** Population structure (K = 2 to 7). *D. cultrate*-5 was the sample used for genome assembly. **(C)** Principal component analysis (PCA) of G1 (group1) to G7 (group7).

Whole-genome SNP data were used to investigate phylogenetic relationships among the 19 accessions. The neighbor-joining (NJ) tree resulted in seven divergent clades: (G1) *D. cana*; (G2) *D. hupeana*, *D. lanceolaria*, and *D. nigrescens*; (G3) *D. dongnaiensis*, *D. oliveri*-1, *D. oliveri*-2, and *D. oliveri*-3; (G4) *D. sissoo*; (G5) *D. odorifera*; (G6) *D. cochinchinensis*-1 and *D. cochinchinensis*-2; and (G7) *D. cultrata*-1, *D. cultrata*-2, *D. cultrata*-3, *D. cultrata*-4, *D. cultrata*-5, *D. cultrata*-6, and *D. cultrata*-7 (**Figure 5A**). Principal component analysis (PCA) agreed well with the NJ tree and showed clear clustering of G1–G7 members (**Figure 5B**) We further analyzed the population structure, and the ADMIXTURE analysis revealed that the data were compatible with seven groups, *K* = 7 (**Figure 5C**). This result was in full agreement with the phylogenetic relationships and PCA results. Notably, as the K value was 3, the G7 group contained seven *D. cultrata* samples but was divided into two subgroups. We assumed that this was because the samples in the two subgroups were not from the same batch. At the same time, the genetic distance between *Dalbergia cultrata* and *Dalbergia cochinchinensis* was smaller than that between *Dalbergia cultrata* and *Dalbergia odorifera* or *Dalbergia sissoo* (**Figure 5**).

### 2.6 Pathways and genes involved in cold stress response

**Figure 6:**
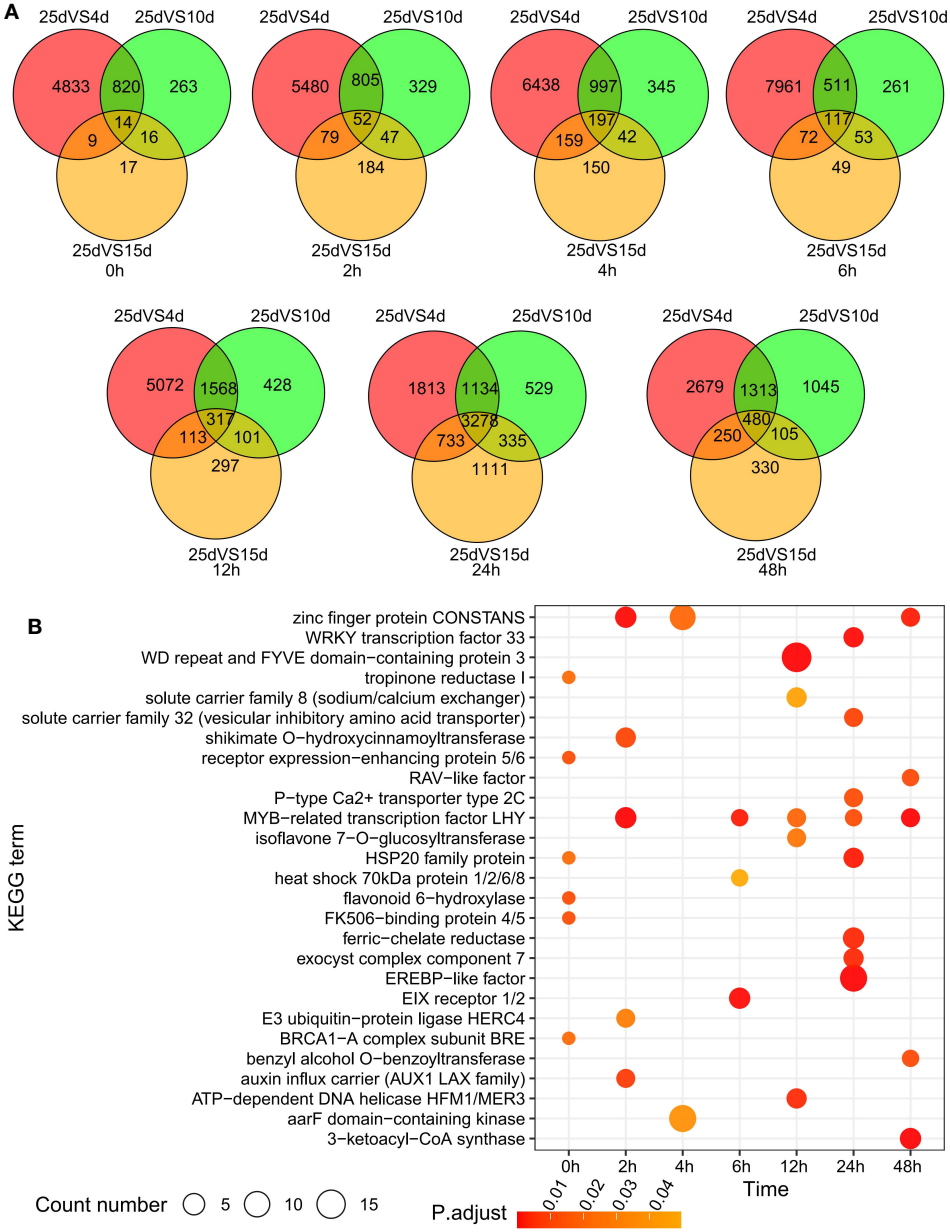
Difference analysis of leaf transcriptome under different temperature and cold stress. **(A)** Differentially expressed genes under low temperature stress from 0 to 48 h. **(B)** KEGG enrichment analysis of differentially expressed genes under low temperature stress from 0 to 48 h.

To analyze the cold resistance mechanism of *D. cultrata* under low-temperature stress, we set up four temperature gradients of 4°C, 10°C, 15°C, and 25°C, of which 25°C was the normal growth temperature, as the control group. The cold stress treatment was conducted for a total of 48 h, and samples were taken at seven time points of 0 h, 2 h, 4 h, 6 h, 12 h, 24 h and 48 h for transcriptome sequencing (**Figure S9**). Differentially expressed genes (DEGs) at various time points were identified using DESeq2 and visualized using a Venn diagram. The number of DEGs shared by the three time points continued to increase from 0 h to 24 h, but gradually decreased from 24 h to 48 h, indicating that *D. cultrata* reached its maximum intensity in response to low temperature within 24 h after being subjected to cold stress, and then gradually adapted to cold stress. At the same time, *D. cultrata* showed an earlier response to cold stress as the intensity of cold stress increased from 15°C to 4°C (**Figure 6A**). We observed that *D. cultrata* completely wilted and failed to return to normal after 48 h of growth at 4°C, whereas it could return to normal growth after 48 h of growth at 10°C and 15°C, and then back to 25°C. KEGG enrichment analysis was performed on the DEGs shared by the three temperatures at each time point. These results revealed that MYB transcription factors were differentially expressed under different cold stress conditions. Many transcription factors or metabolic pathways are related to cold stress, such as zinc finger protein, EREBP-like factor, P-type Ca^2+^ transporter type 2C, and E3 ubiquitin-protein ligase HERC4 (**Figure 6B**).

**Figure 7:**
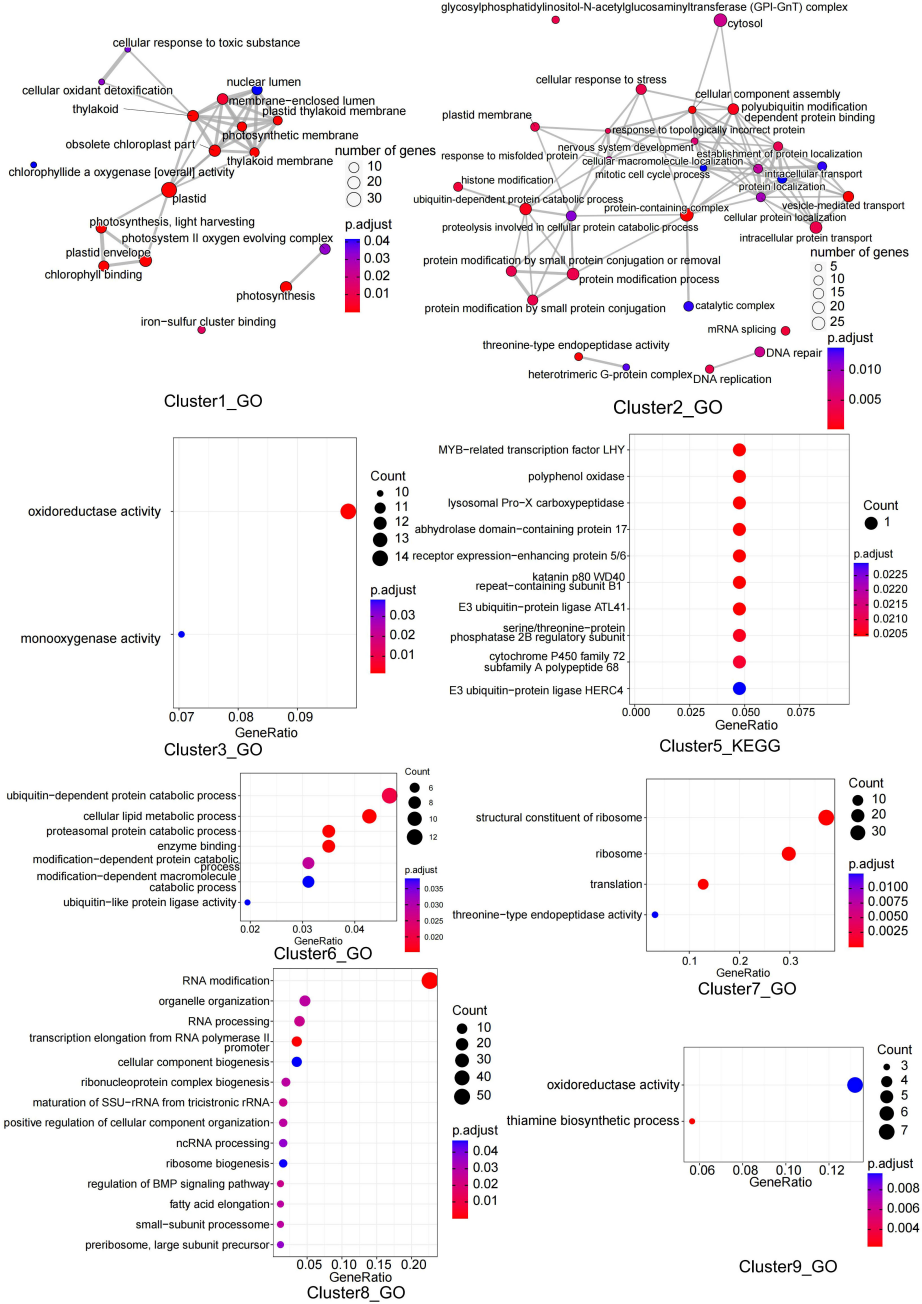
GO or KEGG enrichment analysis of genes in 8 clusters.

The expression trends of the DEGs were analyzed using maSigPro and clustered into nine clusters, with GO and KEGG enrichment analyses performed for each cluster. Cluster1 contained 345 DEGs that were mainly enriched in the photosynthesis pathway and iron-sulfur cluster binding. Cluster2 contained 852 DEGs that were mainly enriched in protein modification, processing, and DNA repair pathways. Cluster3 contained 241 DEGs that were mainly enriched in oxidoreductase activity and monooxygenase activity pathways. Cluster4 contained 777 DEGs; however, no enrichment results were found. Cluster5 contained 35 DEGs, mainly enriched in MYB-related transcription factors, zinc finger protein CONSTANS, E3 ubiquitin-protein ligase, and other pathways. Cluster6 contained 458 DEGs that were mainly enriched in the proteasomal protein catabolic process pathway. Cluster7 contained 132 DEGs, mainly enriched in the structural constituents of ribosomes, translation, and threonine-type endopeptidase activity pathways. Cluster8 contained 390 DEGs that were mainly enriched in RNA processing and RNA modification-related pathways. Cluster9 contained 102 DEGs that were mainly enriched in thiamine biosynthetic processes and oxidoreductase activity pathways (**Figures 7**, **S8**; **Table S7**).

### 2.7 Experimental verification of the expression of key genes

Sixteen genes homologous to nine candidate reference genes found in *Arabidopsis* were identified in the *D. cultrata* genome. Filter out genes showing low expression levels under cold stress and screen the three genes with the lowest coefficient of variation as candidate reference genes for this experiment, namely *Dcu09G016550* (*ACT*), *Dcu10G018280* (*60SrRNA*), and *Dcu09G001470* (*GAPDH*). RT-qPCR verified that the *Dcu09G001470* gene was relatively stable during cold stress. Finally, this experiment used *Dcu09G001470* (*GAPDH*) as the reference gene. RT-qPCR experiments were used to verify the expression levels of eight key genes (**Table S9**) in the low temperature stress pathway, and the correlation between the ΔCt value of RT-qPCR and the FPKM value of transcriptome sequencing was calculated. The correlation was between -0.70 and -0.91, indicating that the results of the low temperature stress transcriptome experiments were accurate (**Figure S10**).

## 3 Discussion

### 3.1 Differences in *Dalbergia* genome size

To date, only *D. odorifera* has been published as a chromosomal-level assembly reference genome in *Dalbergia*, and the genome of *D. cultrata* has not been assessed in previous genome size studies of *Dalbergia* (Hiremath and Nagasampige, 2004). The genome size (690.99Mb) of the *D. cultrata* assembled in this study is close to the result (706.92 Mb) of the *D. cultrata* genome assembled by Illumina short reads (Liu et al., 2022), whereas it is larger than the reported genome size (653.45 Mb) of *D. odorifera* (Hong et al., 2020). There are more expanded genes than contracted genes in the *D. cultrata* genome, which is opposite to the *D. odorifera* genome. This may partly explain why the genome size of *D. cultrata* is larger than that of *D. odorifera*.

### 3.2 Structural variation uncovers a recent inversion

Through genomic structural variation analysis, we found that *D. cultrata* has recently undergone large-scale inversion, which occurred after the separation of *D. cultrata* and *D. odorifera*. The inversion event affects the expression of nearby genes regulating the phenotype and simultaneously reduces the mutation frequency of the genes in the inversion region, resulting in a very high LD of the genes near the inversion. *D. cultrata* genome undergoes an inversion event in the Chr10:43099-9692798 interval, which contains multiple expanded genes. Of these, 8 genes encode cytochrome P450, 7 encode caffeic acid O-methyltransferase, and 4 encode sugar transporters. Cytochrome P450 plays multiple roles in plants, including xenobiotic metabolism, hormones, fatty acids, sterols, cell wall components, biopolymers, and several defense compounds (terpenoids, alkaloids, flavonoids, furan biosynthesis of coumarins, glucosinolates, and allelochemicals) (Pandian et al., 2020). Overexpression of caffeic acid O-methyltransferase 1 enhances melatonin levels and salt stress tolerance in tomato (Sun et al., 2020a).

### 3.3 Metabolic pathway genes are under selection in evolution

Gene *Dcu01G022380* was found to be enriched in the chalcone synthase pathway based on the results of the KEGG enrichment analysis of positively selected genes. Chalcones are rich in plants and are the biogenetic precursors of flavonoids and isoflavones, as well as active lead molecules used to discover new drugs in medicinal chemistry (Rammohan et al., 2020). Fifteen genes involved in the lupeol synthase pathway were annotated in the *D. cultrata* genome. Of these, 14 genes were located in the interval Chr2:76115680-77832057, and seven genes were specific to *D. cultrata*. These may be the key genes for the aroma and medicinal value of *D. cultrata*. Lupeol synthase is a key enzyme involved in lupeol synthesis. Lupeol may be a valuable potential lead compound for the development of anti-inflammatory, antidiabetic, hepatoprotective, and anticancer drugs (Tsai et al., 2016). Many studies have shown that lupeol has great potential for the prevention and treatment of cancers, including liver cancer (Min et al., 2019), lung cancer (He et al., 2011), colorectal cancer (Tarapore et al., 2013), bladder cancer (Prabhu et al., 2016), and osteosarcoma (Zhong et al., 2020; Liu et al., 2021a).

### 3.4 The cold stress regulatory network of *D. cultrata*

The cold stress signal first affected the photosynthetic components. Under cold stress at 4°C and 10°C, photosynthetic elements were always down-regulated from 0 h to 48 h. At a low temperature stress of 15°C, the expression of photosynthetic elements was down-regulated from 0 h to 24 h, and the expression level was not different from that of the control group at 48 h, indicating that it had adapted to the low temperature stress of 15°C at this time. (**Figure S8** Cluster1).

**Figure 8:**
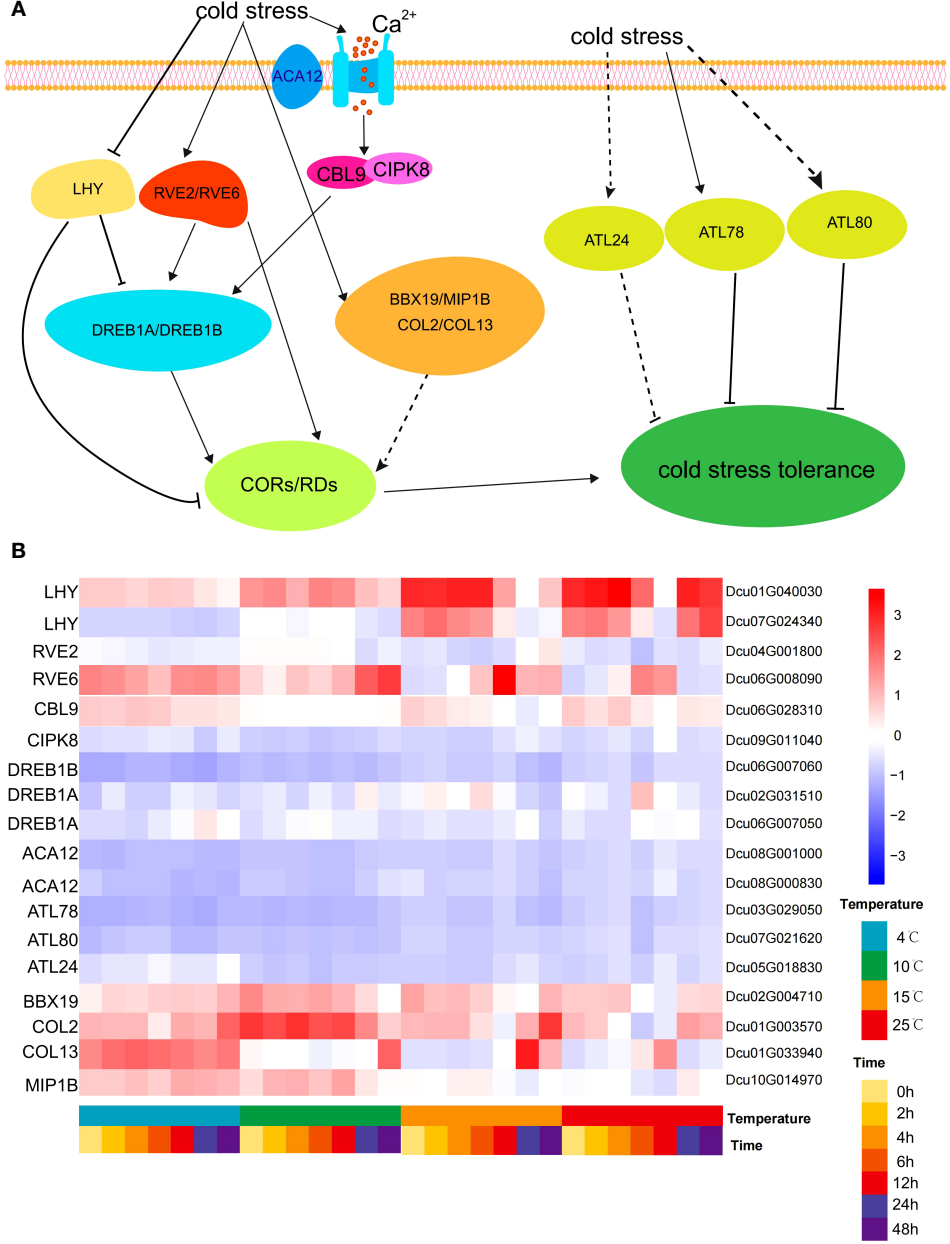
Regulatory network of cold stress. **(A)** Cold stress signaling regulatory network. The cold stress signal opens the Ca^2+^ ion channel through ACA12 located on the plasma membrane to promote the influx of Ca^2+^ into the cell, resulting in the up-regulated expression of CBL9 and CIPK8, which in turn leads to the up-regulated expression of DREB1A/DREB1B. At the same time, the cold stress signal directly stimulates the up-regulation expression of RVE2 and RVE6, and then regulates the DREB gene. DREB gene positively regulates COR/RD gene. RVE2/RVE6 also directly and positively regulate COR/RD genes. At the same time, cold signals negatively regulate LHY transcription factors, which in turn negatively regulate COR/RD genes, and LHY also negatively regulates DREB genes. Cold stress signals can also directly stimulate the up-regulation of ATL24/ATL78/ATL80 genes, and these ATL genes negatively regulate cold stress tolerance. **(B)** Heatmap of the expression of core genes involved in the regulation of cold stress at different temperatures and cold stress at different times.

P-IIB autoinhibitory Ca^2+^-ATPase (ACA) is involved in homeostasis by controlling Ca^2+^ efflux from the cytosol to organelles and/or apoplasts (García Bossi et al., 2019). After 4 h of cold stress, the expression of *ACA12* (*Dcu08G000830, Dcu08G001000*) was upregulated, which promoted Ca^2+^ influx into the cells. Low temperatures trigger plasma membrane stiffening and Ca^2+^ channel activation, leading to an increased Ca^2+^ concentration in the cytosol, which in turn activates Ca^2+^-associated protein kinases. The B-like calmodulin-binding protein (*Arabidopsis CBL9* ortholog *Dcu06G028310*) and CBL-interacting protein kinase (*Arabidopsis CIPK8* ortholog *Dcu09G011040*) were upregulated. CBL proteins are a unique group of calcium sensors in plants that regulate cellular calcium levels by interacting with CIPK (Guo et al., 2018). *CBL1* may cooperate with *CIPK7* to regulate cold signaling in *Arabidopsis* and induce the expression of cold-responsive genes (Huang et al., 2011). CBF1-3 (*CBF1/DREB1B*, *CBF2/DREB1C*, and *CBF3/DREB1A*) in *Arabidopsis* are APETALA2/ETHYLENE-RESPONSIVE (AP2/ERF1)-type transcription factors that directly bind to the conserved CRT/DRE motif in the COR promoter (called the CBF regulon) and activate their expression under cold conditions (Ding et al., 2019). Under normal growth conditions, *LHY* represses *DREB1* expression. Under cold stress conditions, RVE4/RVE6/RVE8 accumulated in the nucleus, and LHY was degraded. Meanwhile, it can be found that the expression level of *LHY* gene decreases from 15°C to 4°C as the intensity of cold stress increases (**Figure 8**). High expression levels of RVE4, RVE6, and RVE8 induce DREB1 gene expression through *cis*-acting evening elements (EEs) (Kidokoro et al., 2022). The RING E3 ligase protein-encoding genes Arabidopsis Tóxicos en Levadura (ATL) 78 and ATL80 are negative regulators of the cold stress response in *Arabidopsis* (Cho et al., 2017). The expression level of the CpBBX19 gene was significantly upregulated after 6 and 12 h of cold treatment in wintersweet (Wu et al., 2021a). After cold stress treatment, BBX19/COL2/COL13/MIP1B encoding zinc finger proteins were upregulated in *D. cultrata*, and these genes may positively regulate the expression of COR/RD genes (**Figure 8**).

## 4 Materials and methods

### 4.1 Materials planting

The samples used for genome survey, assembly, and transcriptome sequencing for genome annotation were collected from the same *Dalbergia cultrata* materials individuals, which were germinated using seeds collected from trees grown in Puer district, Yunnan province, China (22.605 N, 100.639 E). It was planted in the greenhouse of the Forestry Research Institute of the Chinese Academy of Forestry (Beijing, China) for 2 years, and as soon as it was approximately 50 cm in height, sampling was performed. The plant utilized for genome sequencing was identified and confirmed as *Dalbergia cultrata* by Professor Yongqi Zheng, and the voucher herbarium was stored in Research Institute of Forestry, Chinese Academy of Forestry. Related material samples can be obtained by contacting Dr. Ping Huang.

### 4.2 Illumina short-read sequencing

For genome sequencing, fresh young leaves were harvested and immediately frozen in liquid nitrogen for genomic DNA extraction. Genomic DNA was extracted and purified using the Tiangen Extraction Kit (Tiangen Biotech (Beijing) Co., Ltd.). Then, it was fragmented using a Covaris M220 focused ultrasonicator. Illumina PCR-free libraries with insert sizes of 300–500 bp were constructed using the NEBNext Ultra DNA Library Pre Kit for Illumina sequencing. Susequently, 150 bp paired-end sequencing was performed using the Illumina NovaSeq 6000 platform.

### 4.3 Estimation of genome features

Flow cytometry and genome surveys were performed to estimate the genome size of *D. cultrata*. For flow cytometry, cell nuclei suspensions were analyzed using FACSCalibur and the corresponding Cellquest Pro 6.0. The genome size of *D. cultrata* was assessed according to the following formula: GS_unknown_=GS_standard_×PI-fluor_unknown_/PI-fluor_standard_ (GS indicates genome size, PI-fluor indicates the number of red PI fluorescence channels). *Oryza sativa* (389Mb) (Mahesh et al., 2016) and *Glycine max* (1107.4Mb) (Greilhuber and Obermayer, 1997) were used as reference genomes.

Illumina short-read sequences were used for genome surveys. Fastp v0.23.0 was used to filter short reads with default parameters (Chen et al., 2018). K-mer Counter (KMC) v3.0.0 was used to obtain K-mer files from clean data with parameter -k 19 (Kokot et al., 2017). GenomeScope 2.0 was used to estimate the genomic heterozygosity, repeat sequences, and size with parameter -k 19 (Ranallo-Benavidez et al., 2020).

### 4.4 PacBio and Hi-C library construction and sequencing

PacBio library was constructed according to the method of Zhao et al. (Zhao et al., 2022). Briefly, SMRT bell libraries were constructed according to the manufacturer’s protocol, high-quality genomic DNA was purified using the Mobio PowerClean Pro DNA Clean-Up Kit, and DNA quality was assessed by agarose gel electrophoresis. Furthermore, 15–50 kb genomic DNA was sheared and enzymatically repaired. The hairpin adapters were ligated after exonuclease digestion. The resulting SMRT bell templates were size-selected using blue pipin electrophoresis (Sage Sciences). Then, single molecule sequencing was performed on the PacBio RS II platform for the selected size SMRT DNA fragments. The Hi-C library with an insertion size of 300-700bp was constructed according to the method of Rao et al. (Rao et al., 2014). The construction of the Hi-C library mainly includes cell cross-linking, Endonuclease digestion, end repair, cyclization, DNA purification and capture, and computer sequencing.

For Hi-C library construction, young leaves were fixed with formaldehyde and lysed, and then the cross-linked DNA was digested with HindIII restriction enzyme and the 5′ overhangs were biotinylated. After labeling with biotin-14-dCTP, the resulting free blunt ends were ligated. Purified DNA was then treated to remove biotin from the non-ligated DNA ends. For fragmentation, DNA was sheared with a Covaris M220 focused ultrasonicator. The sheared DNA was then repaired, and biotin-containing fragments were isolated using streptavidin beads. A-tailing and adapters were ligated and sequencing libraries were generated. Following library construction, the library's concentration and insert fragment size were determined using Qubit3.0 and GX platforms, respectively. Hi-C library sequenced on an Illumina NovaSeq 6000 platform.

### 4.5 Genome assembly

The primary assembly was performed with PacBio subreads (15-50kb) using CANU (v2.2) (Koren et al., 2017). Based on the assembled genome size, number of contigs, average contig size, N50, assemblies from SMARTdenovo (Liu et al., 2021b), and WTDBG2 (v2.5) (Ruan and Li, 2020), after CANU correction, were selected for merging using quickmerge (v0.3) (Solares et al., 2018) to improve contiguity. Finally, the draft assembly was polished using PacBio long reads with Arrow software and corrected using Illumina paired-end reads with the Pilon (v1.24) software (Walker et al., 2014).

Sequences were mounted on chromosomes according to the method described by Liu et al. (Liu et al., 2020). Hi-C read pairs were aligned to the draft assembly using Juicer V1.06 software (Durand et al., 2016). The resulting contact matrix and draft components were used to construct Hi-C scaffolds using 3D-DNA pipelines (Dudchenko et al., 2017). Finally, LR-Gapcloser (Xu et al., 2019) with PacBio long reads was performed to close the gap, and a pilon was used to polish the assembly with Illumina paired-end short reads. Redundancy was used to eliminate redundancy in unplaced contigs (Pryszcz and Gabaldón, 2016).

### 4.6 Genome annotation

The library of repeat families in our assembled genome was generated using RepeatModeler. RepeatMasker was used to identify repetitive elements based on the repeat library. The prediction and functional annotation of *D. cultrata* protein-coding genes were conducted using the *Sorbus pohuashanensis* genome annotation pipeline (Zhao et al., 2022), integrating homology prediction, *de novo* prediction, and transcriptome prediction. Gene functional annotation was performed using BLAST by searching the Swiss-Prot, TrEMBL, NR, Pfam, and egg-NOG databases. Samples from the roots, branch xylem, branch phloem, young leaves, mature leaves, and petioles were collected for transcriptome sequencing using the standard protocol provided by Oxford Nanopore Technologies (ONT). Illumina NovaSeq 6000 was used for next-generation transcriptome sequencing of the mixed samples. tRNA and rRNA genes were identified using tRNAscan-SE and RNAMMER, respectively. The Rfam database was used to identify non-coding RNAs (ncRNAs) genes.

### 4.7 Analysis of LTR insertion time

The genomes of *A. thaliana, A. nanus, A. hypogaea, A. ipaensis, A. duranensis, C. cajan, C. arietinum, D. odorifera*, *G. soja, G. max, P. trichoca*, and *D. cultrata* (**Table S4**) were used to calculate LTR transposon insertion time.

LTR_FINDER_parallel (Ou and Jiang, 2019) software with default parameters and LTRharvest V1.6.1 software (Ellinghaus et al., 2008) (parameters: -similar 85 -vic 10 -seed 20 -seqids yes -minlenltr 100 -maxlenltr 7000 -mintsd 4 -maxtsd 6 -motif TGCA -motifmis 1) were used to identify full-length LTR repeat retrotransposons (LTR-RTs) in the genome. LTR_retriever V2.9.0 software (Ou and Jiang, 2018) (parameters:-u 7e-9) was used to combine the LTR-RTs identified by LTR_finder_parallel and LTRhavest, and calculate the LTR insertion time. The molecular clock r value was selected 7 * 10^-9^ by set the LTR_retriever parameter -u 7e-9 to calculate the LTR insertion time (Ossowski et al., 2010).

### 4.8 Quality assessment of genome assemblies

Three methods were used to assess the quality of assembled genomes: Illumina read alignment, BUSCO evaluation, and whole-genome high long terminal repeat (LTR) assembly index (LAI) score evaluation. The LTR_retriever was also used to calculate the LAI value of the genome and LTR insertion time. Genome integrity was assessed using the Embryophyta plant database of BUSCO v5.2.1 (Manni et al., 2021) containing 1,614 conserved core genes. Consensus quality value (QV) of Illumina short reads were calculated using Merqury v1.3 (Rhie et al., 2020). The integrity of the genome assembly was assessed using CEGMA v2.5 (Parra et al., 2007), which contains 458 conserved core eukaryotic genes.

### 4.9 Genome gene duplication analysis

The stricter version of DupGen_finder (Qiao et al., 2019) unique with default parameters, was used to identify *D. cultrata* genome genes derived from different modes of gene duplication: WGD, TD, PD, TRD, and DSD. KaKs_Calculator v2.0 software (Wang et al., 2010) was used to calculate Ka, Ks, and Ka/Ks values of gene pairs. The proportion of each homologous gene to the 4DTv site was calculated using Perl script (https://github.com/JinfengChen/Scripts/blob/master/FFgenome/03.evolution/distance_kaks_4dtv/bin/calculate_4DTV_correction.pl).

### 4.10 Gene family classification

Gene family cluster analysis was performed on the protein sequences of 16 species (*A. thaliana, A. nanus, A. hypogaea, A. ipaensis, A. duranensis*, *C. cajan*, *C. arietinum*, *D. odorifera*, *G. soja*, *G. max*, *V. vinifera*, *Lupinus angustifolius*, *Medicago truncatula*, *P. trichoca*, Spatholobus suberectus, and *D. cultrata*) using Orthofinder v2.4 software (Emms and Kelly, 2019) (diamond, E-value 0.001). In total, 702 genes were identified as single-copy genes, covering at least 75% of the 16 species. PANTHER V15 database (Mi et al., 2018) was used to annotate the obtained gene families.

### 4.11 Phylogenetic analysis

A phylogenetic tree was constructed and the divergence time was estimated according to Zhao et al. (Zhao et al., 2022). The 702 single-copy genes described above were used to construct a phylogenetic tree, and *V. vinifera* was used as an outgroup for the root tree. Using TimeTree (http://www.timetree.org/), the divergence times were estimated as follows: *V. vinifera* vs. *G. max* at 107–135 MYA, *C. cajan* vs. *G. max* at 11.7–27.5 MYA, *V. vinifera* vs. *D. odorifera* at 107–135 MYA, *D. odorifera* vs. *A. ipaensis* at 26–51 MYA, *A. ipaensis* vs. *A. nanus* at 53–85 MYA, *P. trichocarpa* vs. *A. ipaensis* at 101–131 MYA.

The CAFE v4.2 software (Han et al., 2013) used the results of phylogenetic tree with divergence time and gene family clustering to predict the expansion and shrinkage of the species’ gene families relative to their ancestors.

### 4.12 Positive selection analysis

Previously identified single-copy gene families of *A. hypogaea*, *A. ipaensis*, *D. odorifera*, and *A. nanus* were used. Each gene family was analyzed using MAFFT (parameters: –localpair –maxiterate 1000) to align protein sequences and PAL2NAL to reverse codons to align sequences. Positively selected genes were identified using the CodeML module of PAML (F3 × 4 model using codon frequencies).

### 4.13 Collinearity and WGD analysis

The genomes of *A. thaliana, D. odorifera, D. cultrata, P. trichocarpa, V. vinifera, A. ipaensis*, and *A. duranensis* genomes, which are evolutionarily closely related to *D. cultrata*, were used for collinearity analysis. The protein and CDS sequences of the species were compared using Diamond (v0.9.29.130) (Buchfink et al., 2015) (parameter: e<1e−5) to identify similar gene pairs. The C-score value was used to filter the blast results using JCVI v0.9.13 (Tang et al., 2015) (parameter: C-score>0.5). R packages ggalluvial (V0.12.3) (Brunson, 2020) was used to draw a collinearity picture of the linear patterns of each species. WGDI (V0.58) (Sun et al., 2022) was used to analyze Ks values for collinear gene pairs and a Perl script was used to analyze 4DTV values.

### 4.14 Genome structure variation analysis

AnchorWave (V1.0.1) (Song et al., 2022) was used to identify the collinear regions of *D. odorifera* vs. *D. cultrata, Arachis duranensis* vs. *D. odorifera*, *Arachis duranensis* vs. *D. cultrata*. R was used to visualize the collinearity of genome output by Anchorwave. SYRI v1.6.1 (Goel et al., 2019) was used to identify the structural variation and collinearity in the *D. cultrata* and *D. odorifera* genomes. Plotsr v0.5.4 (Goel and Schneeberger, 2022) was used to visualize the structural variation information output by the SYRI.

### 4.15 Collection of sequence data, sequence alignment, and SNP identification

In this study, *Dalbergia hupeana*, *D. cochinchinensis*, *D. sissoo*, *D. odorifera*, and *D. cultrata* were selected for DNA isolation from the leaf tissues of each accession using a Plant DNA Mini Kit (Aidlab Biotech) and high-throughput sequencing (**Table S8**). DNA libraries with 350-bp inserts were constructed for each accession using the Illumina NovaSeq 6000 platform following the manufacturer’s specifications, and 125-bp paired-end reads were generated. Additional sequences of ten accessions were downloaded from the NCBI database with the corresponding biological project number PRJEB49228. The accession numbers are listed in **Table S8**.

### 4.16 Read alignment and variation calling

Fastp v0.23.0 (Chen et al., 2018) was used to filter raw data and obtain clean data. Clean reads were aligned to the reference genome of *D. cultrata* using BWA software (v0.7.17). BAM alignment files were generated using the SAMtools software (v1.9) (Li et al., 2009). SNPs were identified using the software GATK (v4.1.3.0) (DePristo et al., 2011), and the following parameters were used for filtering SNPs and Indels: ‘QD < 2.0 || MQ < 40.0 || FS > 60.0 || SOR > 3.0 || MQRanksum < -12.5 || ReadPosRanksum < -8.0’ and ‘QD < 2.0 || FS > 200.0 || SOR > 10.0 || InbreedingCoeff < -0.8 || ReadPosRanksum < -20.0’.

### 4.17 Phylogenetic tree and population structure

SNPs were used to calculate genetic distances between individuals. An individual-based neighbor-joining (NJ) tree was constructed using the p-distances model in Phylip (v3.697) and visualized using software MEGA5. The population genetic structure was determined using ADMIXTURE software (v1.3.0). The assumed number of clusters (*K*) was set from 2 to 10, with 10,000 iterations per run. Principal component analysis of the SNPs was performed using GCTA software (v1.91.5) (Yang et al., 2013).

### 4.18 Seedlings of *D. cultrata* treated with low temperature stress

To study the effect of low temperature on the growth and development of *D. cultrata*, we used seedlings at the developmental stage (three years old) to conduct low-temperature stress experiments. In September 2022, the plants were cultivated in a constant temperature light incubator, and the light/dark time cycle as 14h/10h, 07:00am every day as the starting time of light, and the growth temperature was set at 25°C. At 09:30 on September 7, 2022, the temperature of three of the incubators was lowered to 4°C, 10°C, and 15°C for cold stress treatment, and the other incubator was kept at 25°C as the control group and lowered to the corresponding temperature, taking the first sample at, and recording this time as 0 h. Starting from 0 h, samples were taken at 2 h, 4 h, 6 h, 12 h, 24 h, and 48 h thereafter. There were nine plants at each temperature, and the leaves of the same leaf position for each of the three plants were mixed as a biological replicate, and each sample had three biological replicates (**Figure S9**). Immediately after sampling, leaves were quickly frozen in liquid nitrogen for subsequent RNA extraction.

### 4.19 RNA library construction and sequencing

Total RNA was extracted from leaf samples preserved in liquid nitrogen using an RNAprep Pure Plant Kit (Tiangen DP441), and genomic DNA contamination was removed using DNase I (Tiangen). RNA degradation and contamination was monitored on 1% agarose gels. RNA purity was checked using the NanoPhotometer^®^ spectrophotometer (IMPLEN, CA, USA). RNA concentration was measured using Qubit^®^ RNA Assay Kit in Qubit^®^3.0 Flurometer (Life Technologies, CA, USA). RNA integrity was assessed using the RNA Nano 6000 Assay Kit of the Agilent Bioanalyzer 2100 system (Agilent Technologies, CA, USA). The isolated 1 µg RNA was used for cDNA library construction using the NEBNext Ultra RNA Library Preparation Kit for Illumina (New England Biolabs, Ipswich, MA, USA), with fragment lengths of approximately 150 bp. The cDNA library was paired-end sequenced using an Illumina NovaSeq 6000 platform.

### 4.20 Transcriptome analysis

Fastp v0.23.0 (Chen et al., 2018) was used to filter raw data and obtain clean reads. The filtered reads were mapped to the reference genome of *D. cultrata* using Hisat2 v2.1.0. The read count and the level of gene expression were quantified using the featureCounts v2.0.1 program (Liao et al., 2014). The 25°C sample at each time point was used as the control group, and the 4°C, 10°C, and 15°C samples were compared with the 25°C sample. The differentially expressed genes were then measured using the DESeq2 program (Love et al., 2014), with the following criteria: FDR < 0.01 and absolute fold change >1. At the same time, edgeR 3.36.0 version (Robinson et al., 2010) was used to normalize the obtained expression matrix, and the R package maSigPro 1.66.0 version (Nueda et al., 2014) was used to analyze the trend of differentially expressed genes. The hclust method was used to cluster the differentially expressed genes into nine clusters, and then each GO and KEGG enrichment analysis was performed on the genes of each cluster.

### 4.21 Gene enrichment analysis

All GO and KEGG enrichment analyses were performed using clusterProfiler v4.2.2 (Wu et al., 2021b). The enrichment analysis used the default parameters, and the top20 terms are shown in the figure.

### 4.22 Selection of reference genes for *D. cultrata*

Nine genes (*ACT, TUA, TUB, GAPDH, EF-1γ, UBQ, UBC, 60S rRNA*, and *eIF6A*) were used as candidate reference genes following the method of Wang et al. (Wang et al., 2019). The coding sequences (CDS) corresponding to these genes in the Araport11 version were downloaded from the TAIR database (https://www.arabidopsis.org/). These sequences were then compared with the CDS sequences of all genes in *D. cultrata* in a local database using Blastn to identify homologous genes. To determine the most stable expression and minimize variability, the coefficient of variation was calculated for the expression levels of these internal reference genes at all temperature periods using the FPKM values from the transcriptome data under cold stress. The internal reference genes with the lowest coefficient of variation were chosen for further analysis.

### 4.23 Real-time quantitative reverse transcription PCR experiment and analysis

The cDNA obtained from reverse transcription of the cold stress transcriptome experiment was used as the substrate template for RT-qPCR amplification. The key genes in the cold stress pathway (**Figure 8B**) and the screened internal reference genes were selected, and quantitative primers were designed using the Primer3 online tool (https://primer3.ut.ee/), and the primer sequences were finally used in **Table S9**. Then, the RT-qPCR fluorescence quantitative kit from SYBR^®^ Green Realtime PCR Master Mix (TOYOBO, Japan) was used to experiment according to the official instructions. The original Ct values were converted into relative expression levels ΔCt (ΔCt = key gene Ct value - reference gene Ct value) using the 2-ΔΔCt method (Livak and Schmittgen, 2001). The Pearson correlation coefficient between the ΔCt value of each key gene and the corresponding FPKM value of the transcriptome was computed and visualized using the ggpubr package in the R language.

## Data availability statement

Raw data for genome assembly, annotation, resequencing, and transcriptome analysis were uploaded to the NCBI SRA database under the Bioproject ID: PRJNA854315. Illumina data SRR19913573 for D. cultrata genome size survey. Data for genome assembly: PacBio data SRR19913575 and Hi-C data SRR19913574. For encoding gene prediction annotations, Illumina data SRR22795462 and ONT data SRR19909638-SRR19909643. Resequencing data for other species of Dalbergia SRR19970616-SRR19970623. Cold stress transcriptome data SRR22198013-SRR22198096. Genome annotations are deposited in FigShare (https://doi.org/10.6084/m9.figshare.20222340). Whole genome sequence data have been deposited in the Genome Warehouse at the National Genomics Data Center (Chen et al., 2021; Members and Partners, 2021), under accession number GWHBJUT00000000, which is publicly accessible at https://ngdc.cncb.ac.cn/gwh. Resequencing data for Thailand were downloaded from the NCBI SRA database ERP133710. The original flow cytometry data were stored in the flow repository database (https://flowrepository.org/), and the access number was FR-FCM-Z6YZ.

## Author contributions

PH: Conceptualization, Writing-Original Draft. CL: Data Curation, Validation. FL: Revising -Original Draft, Resources. YL: Data Curation, Investigation. YiZ: Resources. BL: Supervision. YoZ: Conceptualization, Writing - Review & Editing, Funding acquisition. All authors contributed to the article and approved the submitted version.
