# Supplementary material for: Chromosome-level genome assembly and population genetic analysis of a near-threatened rosewood species (Dalbergia cultrata Pierre Graham ex Benth) provide insights into its evolutionary and cold stress responses
Source: Front Plant Sci. 2023 Sep 21;14:1212967. doi: 10.3389/fpls.2023.1212967 (PMC10552272; doi:10.3389/fpls.2023.1212967)
Supplement: Supplementary file 1 [file DataSheet_1.zip › Supplemental Figures.docx]

# Supplemental Figures


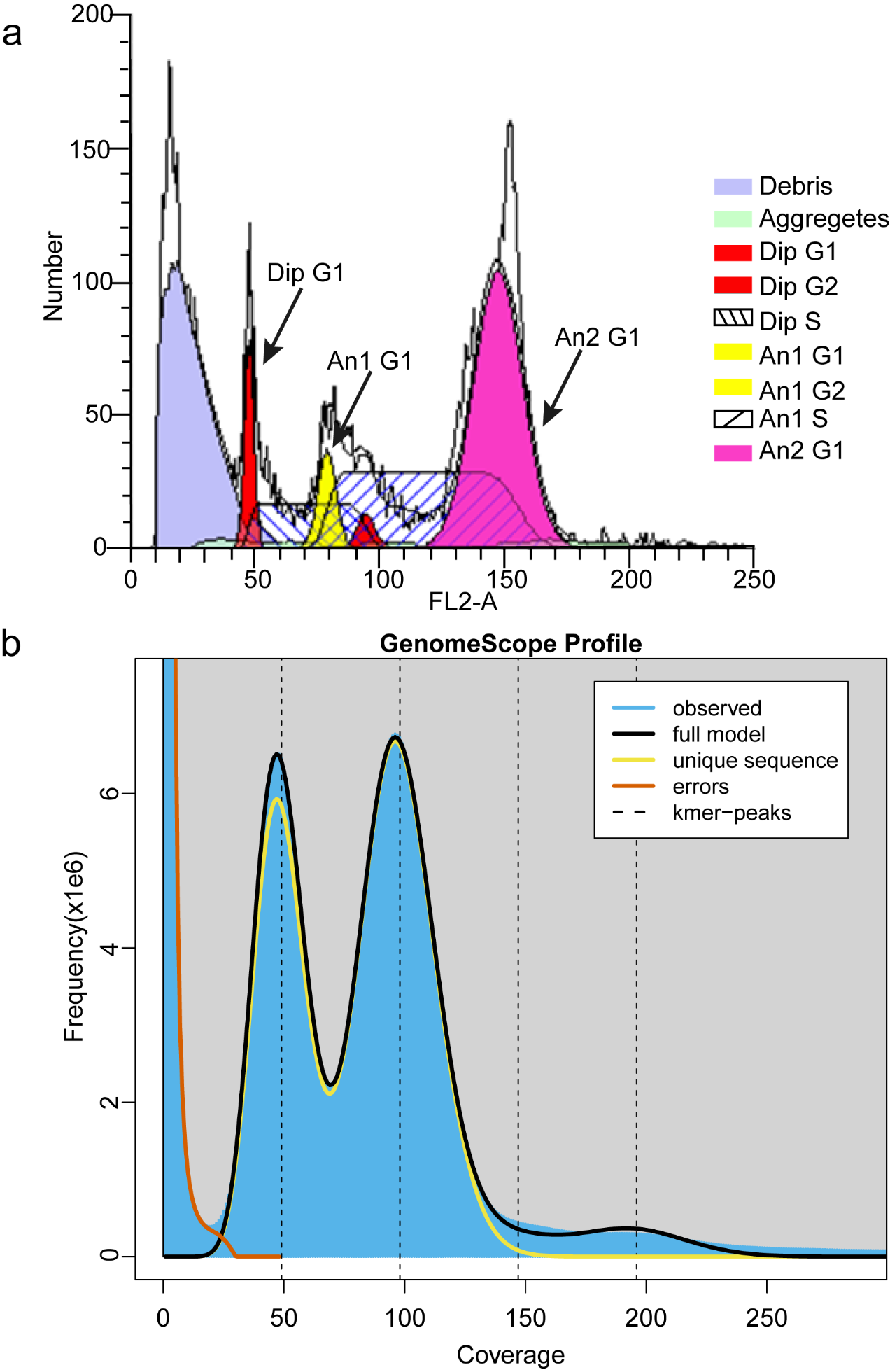


## FigS1. Genome Size Assessment

a. Flow cytometry to estimate genome size. Dip (*Oryza sativa*) G1: 28.75 % at 47.69, An1 (*Dalbergia cultrata*) G1: 12.90 % at 78.94, An2 (*Glycine max*) G1: 100.00 % at 147.59. **b**. The 19-mer distribution of Illumina short reads in *D. cultrata*. The x-axis shows the frequency or the number of times a given k-mer (k-mer depth). The y-axis shows the total number of k-mers with a given frequency (a given depth). Two peaks (blue line) were observed indicating heterozygosity in *D. cultrata*.


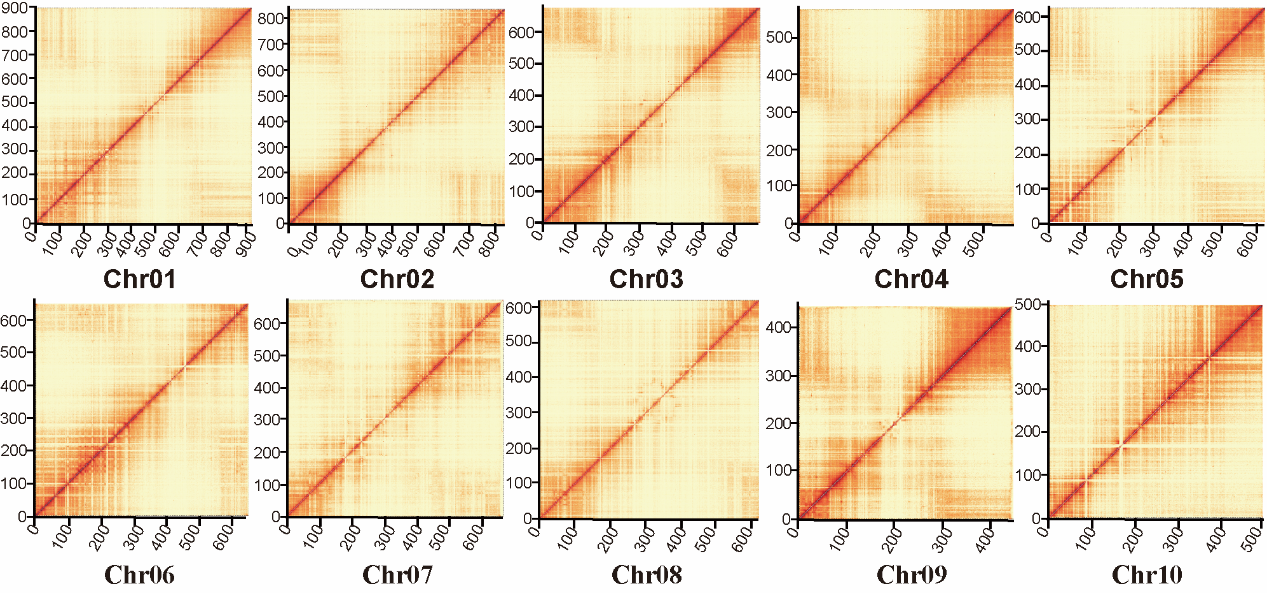


## FigS2. Hi-C interaction heat map


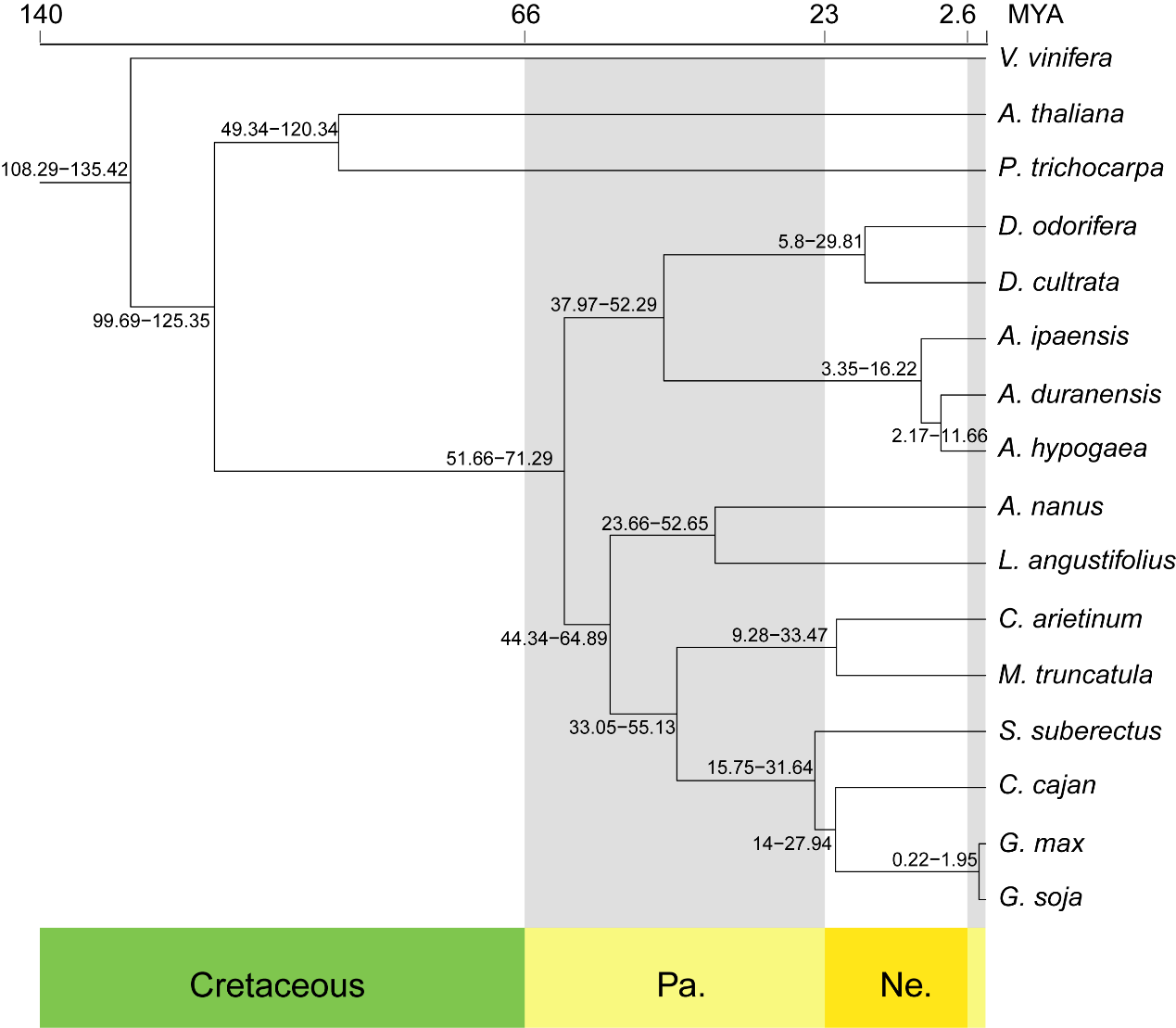


## FigS3. Evolutionary time tree of 16 species


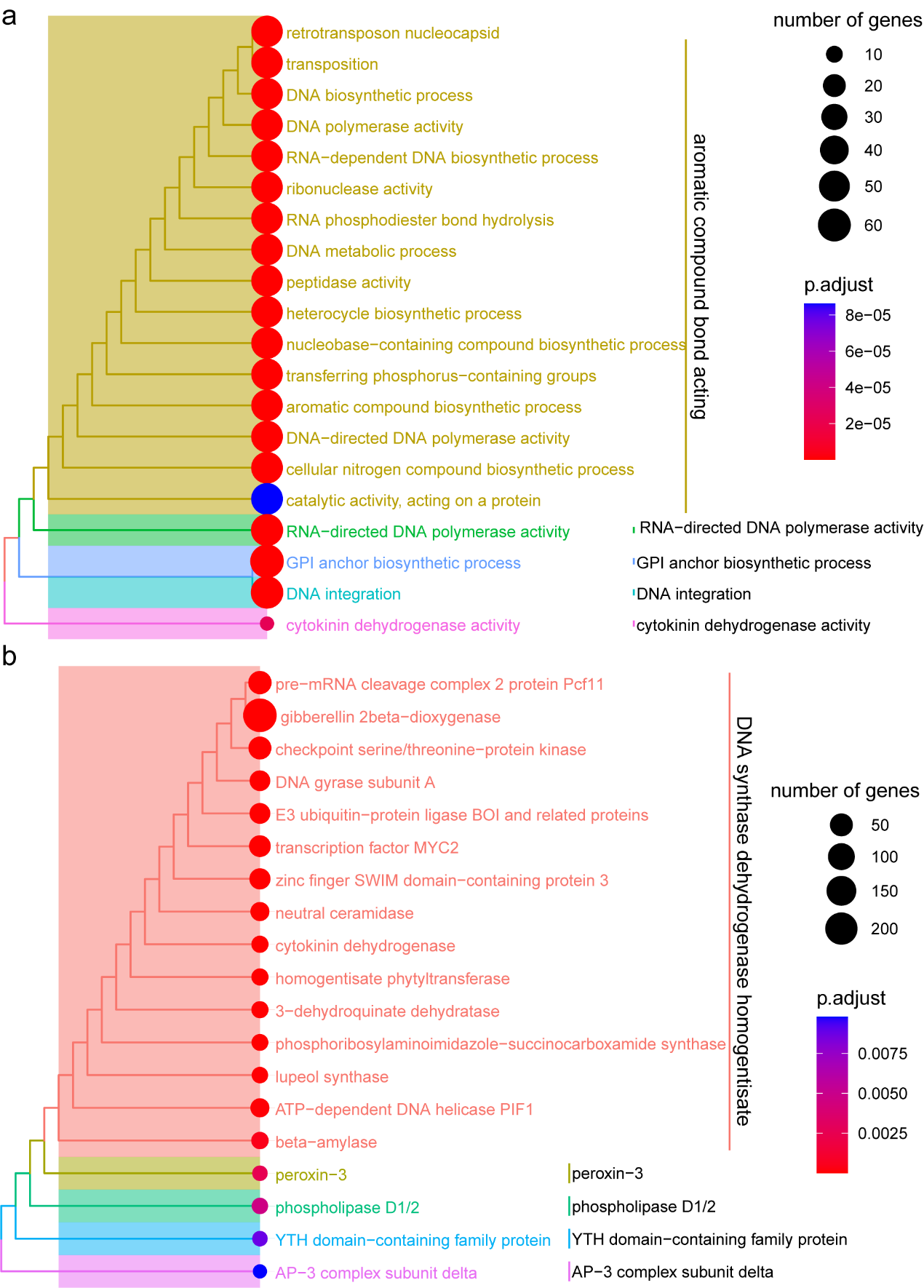


## FigS4. Specific gene GO and KEGG enrichment


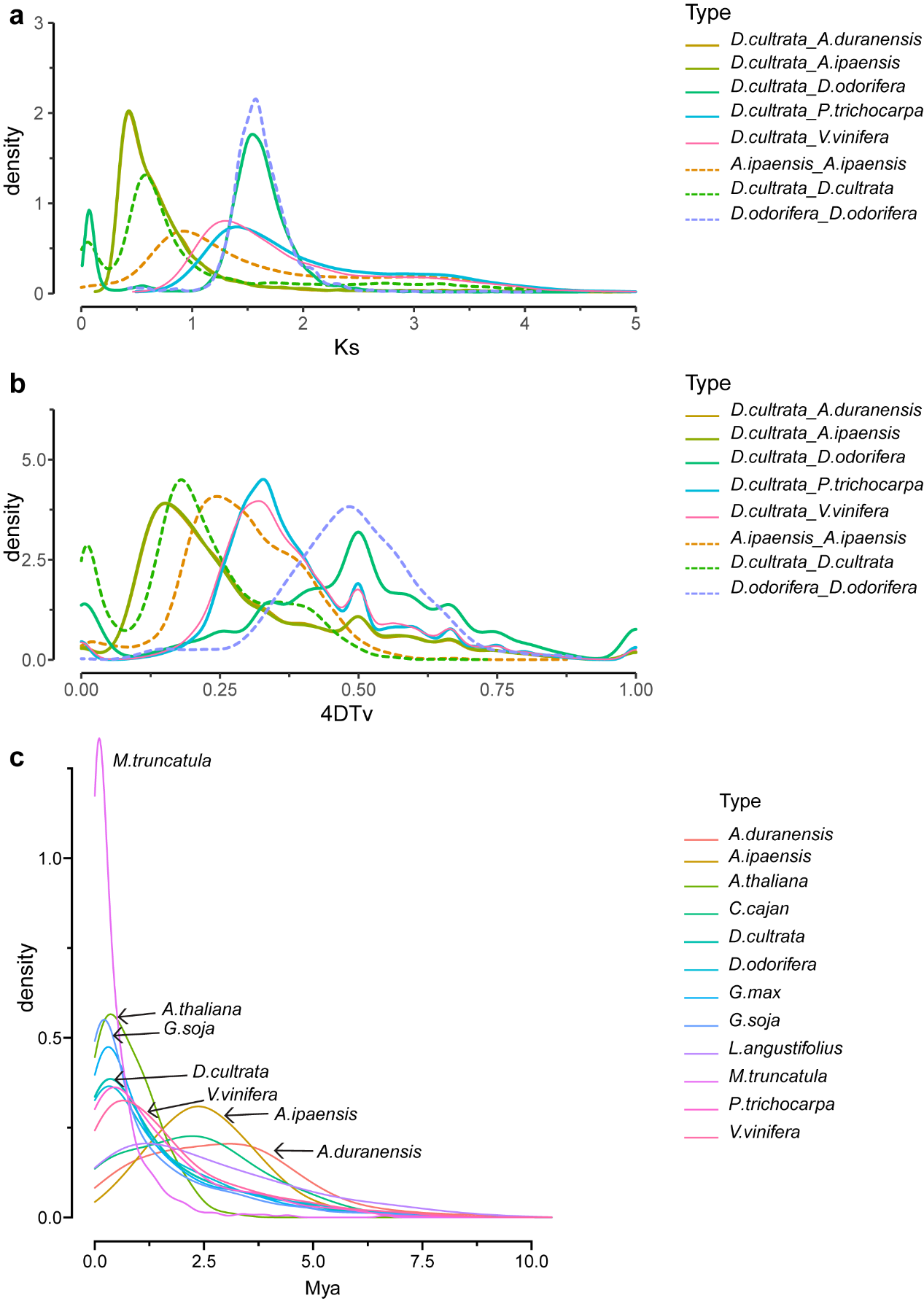


## FigS5. Distribution of Ks, 4DTv and ages of LTR of *D. cultrata* and other species

**a.** Ks distribution of *D. cultrata* and other representative species; **b.** 4DTv distribution of *D. cultrata* and other representative species; **c.** Ages of LTR of *D. cultrata* and other species (Molecular clock r is 7*10^-9^).


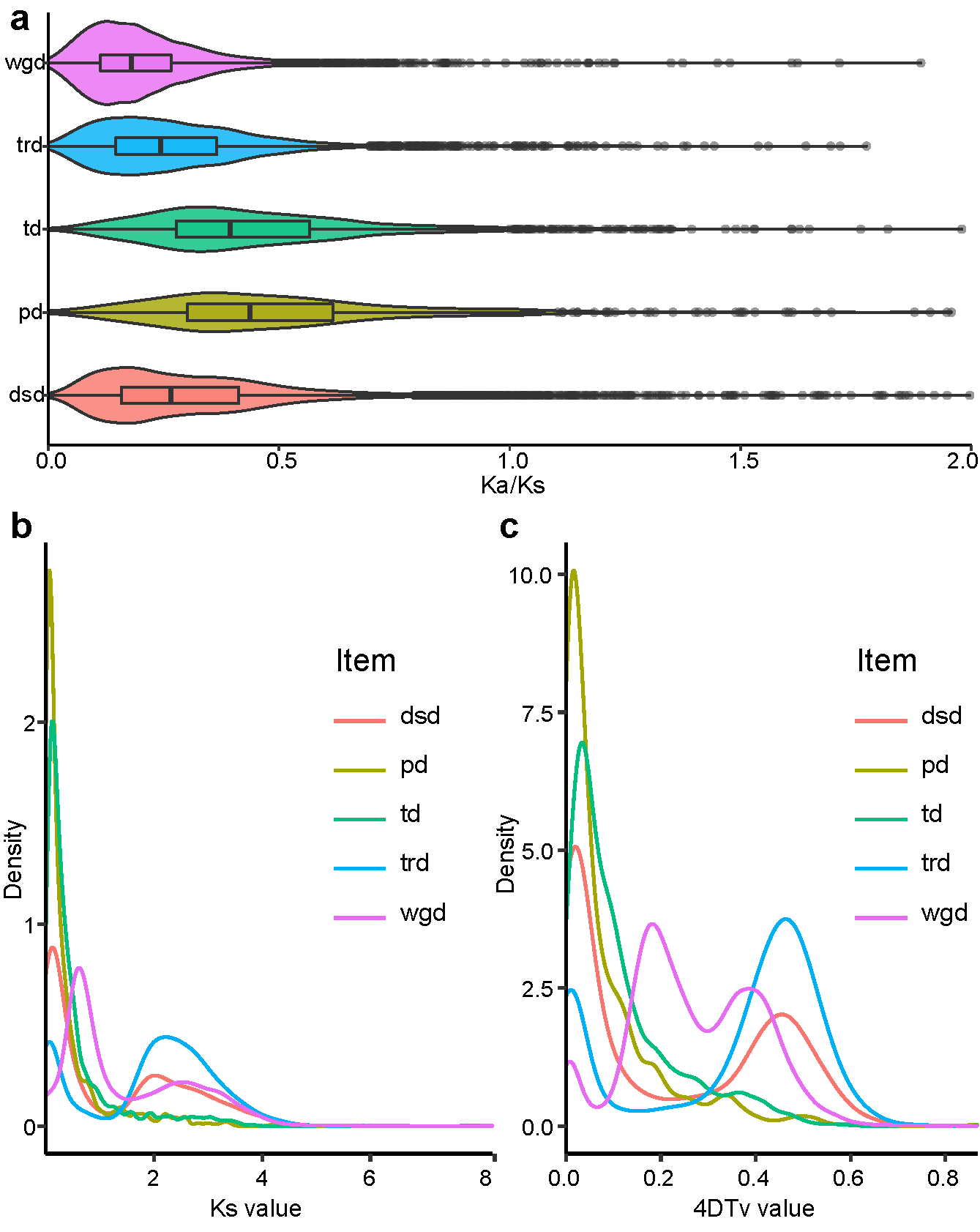


## FigS6. Gene duplication and evolution

**a**. Distribution of Ka/Ks of 5 replication types; **b**. Distribution of Ks of 5 replication types; **c**. Distribution of 4DTv of 5 replication types.


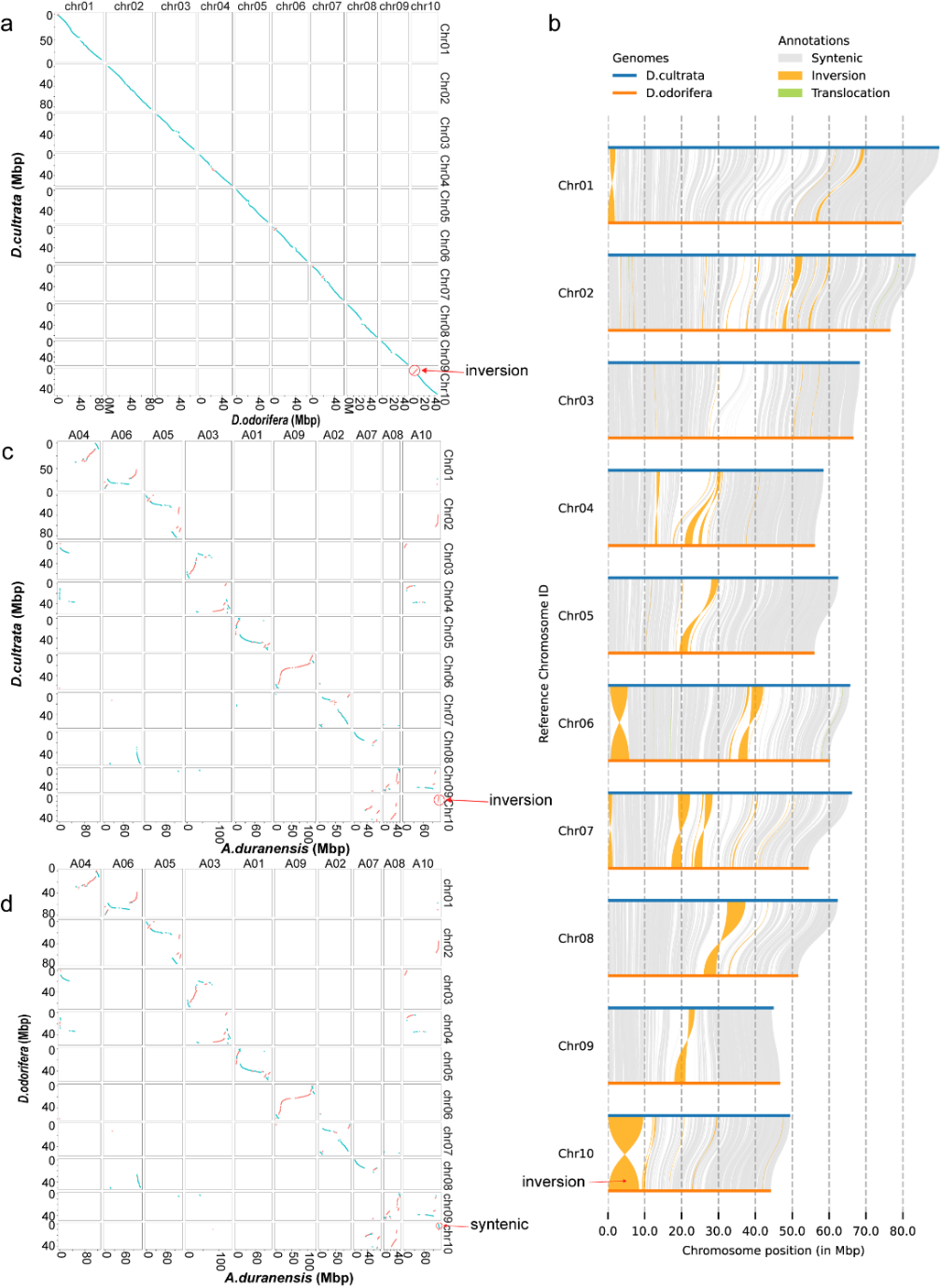


## FigS7. Genome structure variation

a. *D. odorifera* vs. *D. cultrate*. b. All structural variation in the genome *D. odorifera* vs. *D. cultrate*. c. *D. cultrate* vs. *A. duranensis.* d. *D. odorifera* vs. *A. duranensis.* Each point in panels a, c, d is plotted against the starting coordinates of the reference genome and the query genome for each anchor point. The same strand is shown in blue and inverted in red.


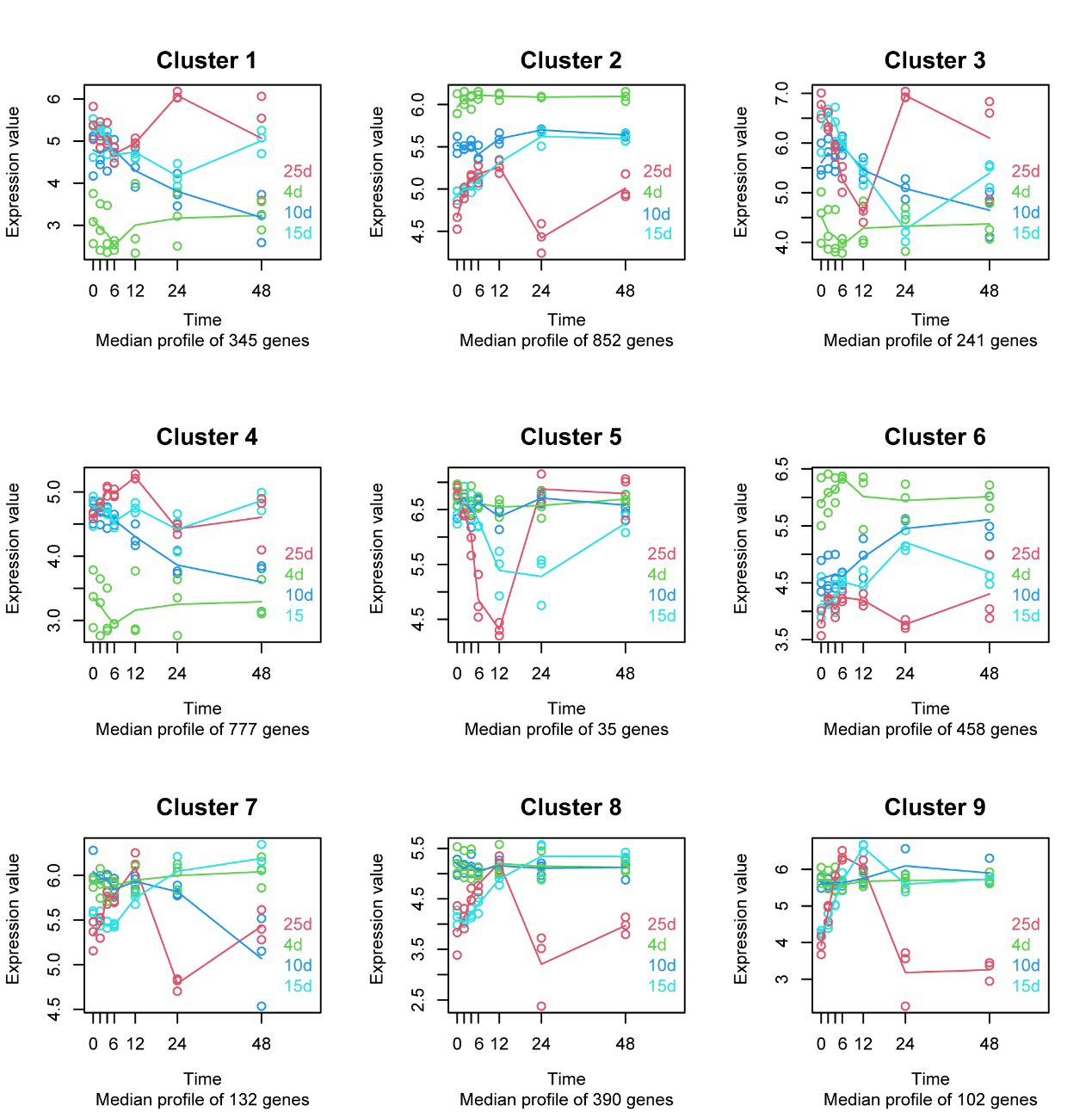


## FigS8. The expression trend graph of 9 clusters of differentially expressed genes in the transcriptome


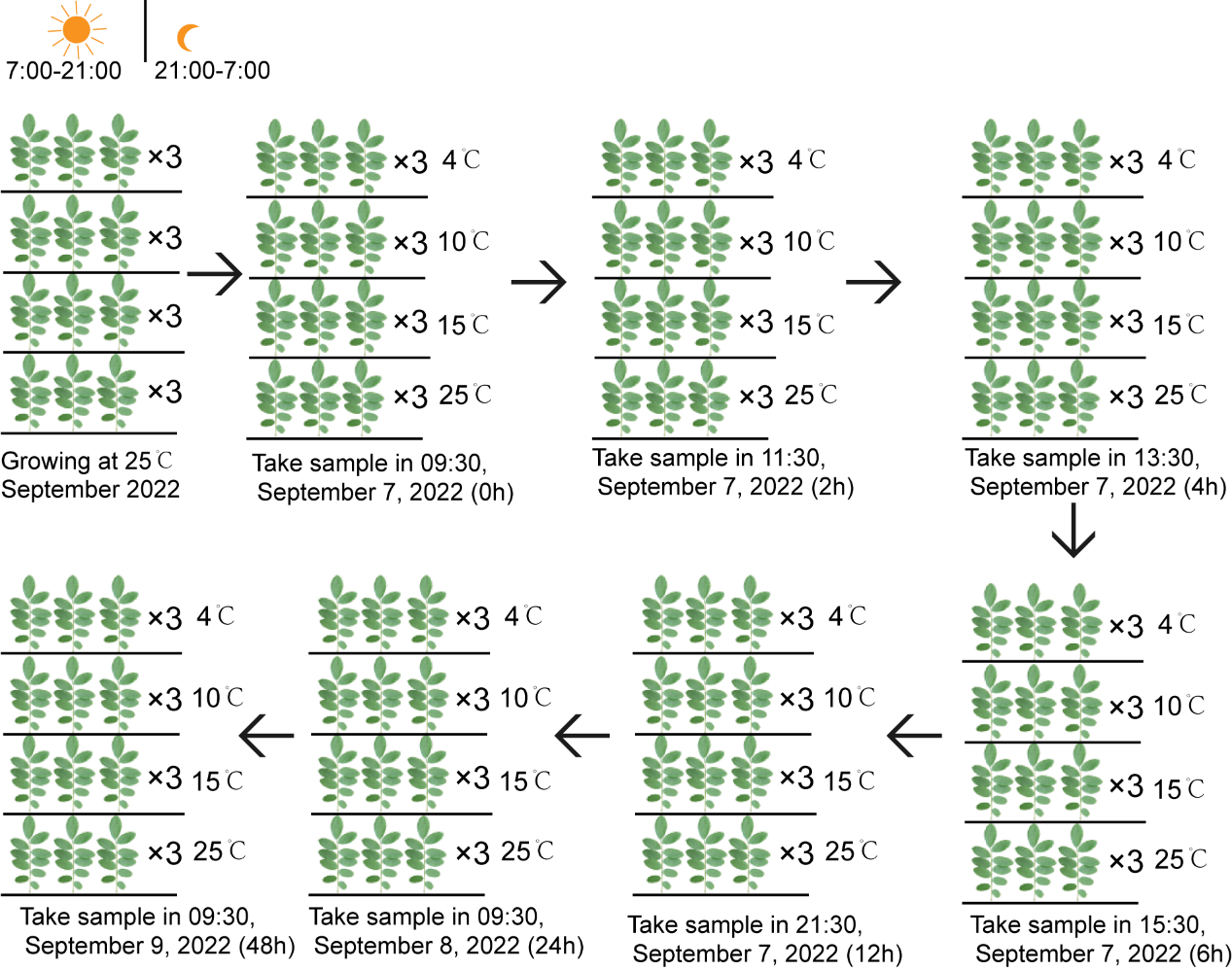


## FigS9. Low temperature stress transcriptome experiment design pattern diagram


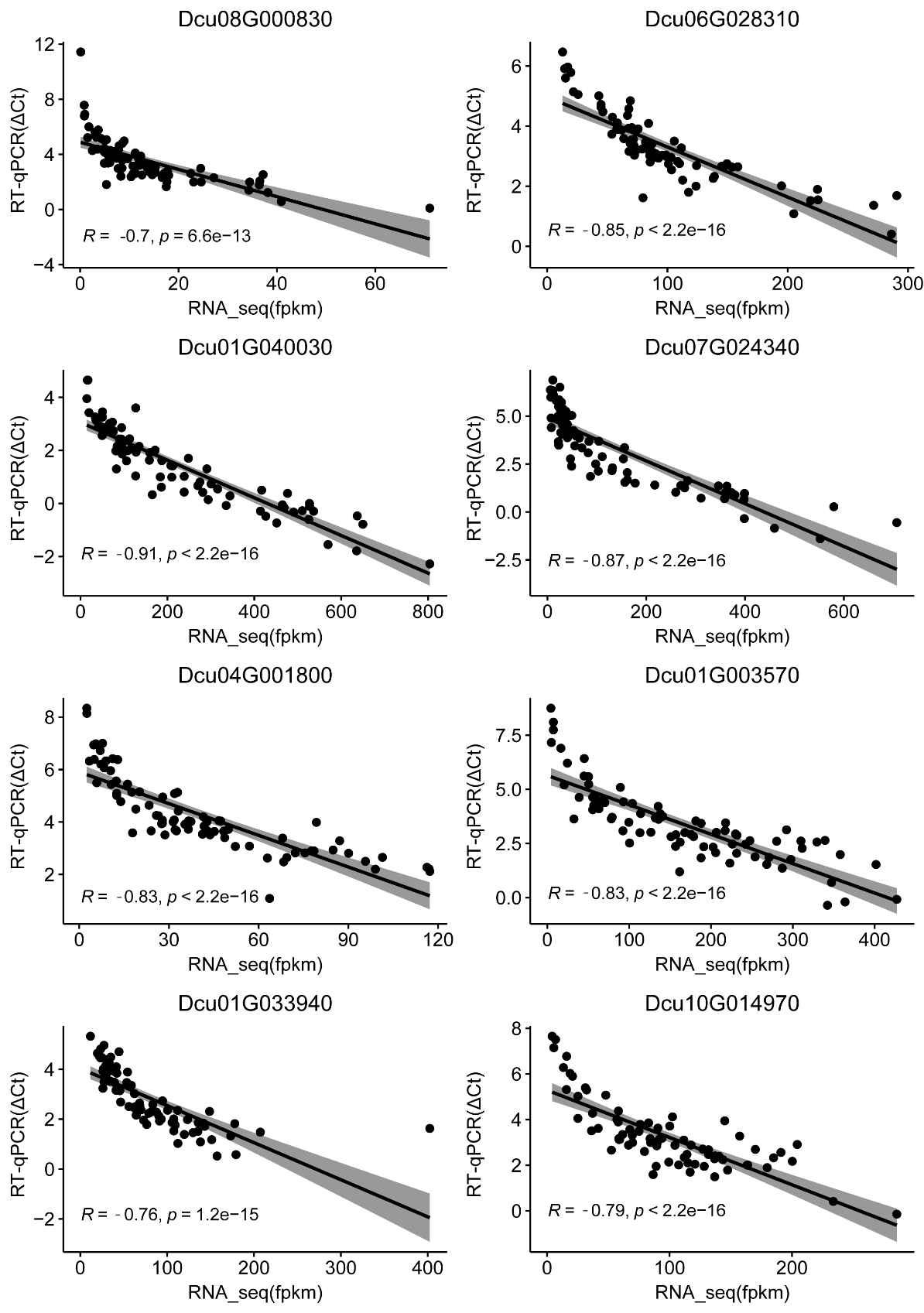


## FigS10. Correlation analysis between RT-qPCR results and transcriptome expression of key genes under low temperature stress (*Dcu09G001470* (GAPDH) as RT-qPCR reference gene)
