# Supplementary material for: Chromosome-level genome assembly and population genetic analysis of a near-threatened rosewood species (Dalbergia cultrata Pierre Graham ex Benth) provide insights into its evolutionary and cold stress responses
Source: Front Plant Sci. 2023 Sep 21;14:1212967. doi: 10.3389/fpls.2023.1212967 (PMC10552272; doi:10.3389/fpls.2023.1212967)
Supplement: Supplementary file 1 [file DataSheet_1.zip › Supplemental Tables.docx]

# Supplemental Tables

## Table S1. BUSCO evaluation result

| Type | Genome | Protein |
| --- | --- | --- |
| Complete BUSCOs | 1592 (98.60%) | 1557 (95.50%) |
| Complete and single-copy BUSCOs | 1501 (93.00%) | 1462 (90.60%) |
| Complete and duplicated BUSCOs | 91 (5.60%) | 95 (5.90%) |
| Fragmented BUSCOs | 9 (0.60%) | 32 (2.00%) |
| Missing BUSCOs | 13 (0.80%) | 25 (1.50%) |
| Total BUSCO groups searched | 1614 | 1614 |

## Table S2. Repeat sequence

| Type | Number | Length | Rate(%) |
| --- | --- | --- | --- |
| ClassI:Retroelement | 413283 | 301645708 | 43.65 |
| ClassI/DIRS | 515 | 27793 | 0 |
| ClassI/LINE | 48598 | 11328020 | 1.64 |
| ClassI/LTR/Cassandra | 31 | 5210 | 0 |
| ClassI/LTR/Caulimovirus | 1814 | 1126623 | 0.16 |
| ClassI/LTR/Copia | 81916 | 60472599 | 8.75 |
| ClassI/LTR/ERV | 9223 | 1055294 | 0.15 |
| ClassI/LTR/Gypsy | 143240 | 172093505 | 24.91 |
| ClassI/LTR/Pao | 2388 | 300882 | 0.04 |
| ClassI/LTR/Unknown | 121968 | 54777175 | 7.93 |
| ClassI/LTR/Viper | 41 | 5567 | 0 |
| ClassI/SINE | 3549 | 453040 | 0.07 |
| ClassII:DNA transposon | 262124 | 62486530 | 9.04 |
| ClassII/Academ | 47 | 4982 | 0 |
| ClassII/CACTA | 18328 | 4802070 | 0.69 |
| ClassII/Crypton | 866 | 54152 | 0.01 |
| ClassII/Dada | 380 | 50235 | 0.01 |
| ClassII/EnSpm | 89 | 45510 | 0.01 |
| ClassII/Ginger | 298 | 15609 | 0 |
| ClassII/Helitron | 7292 | 2940563 | 0.43 |
| ClassII/IS3EU | 298 | 18241 | 0 |
| ClassII/Kolobok | 589 | 45379 | 0.01 |
| ClassII/MITE | 1523 | 598452 | 0.09 |
| ClassII/Maverick | 543 | 30075 | 0 |
| ClassII/Merlin | 144 | 8362 | 0 |
| ClassII/Mutator | 19000 | 5030664 | 0.73 |
| ClassII/Novosib | 516 | 27732 | 0 |
| ClassII/P | 427 | 22817 | 0 |
| ClassII/PIF-Harbinger | 11402 | 2445962 | 0.35 |
| ClassII/PiggyBac | 600 | 35604 | 0.01 |
| ClassII/Sola | 289 | 17292 | 0 |
| ClassII/Stowaway | 203 | 43703 | 0.01 |
| ClassII/Tc1-Mariner | 3947 | 280147 | 0.04 |
| ClassII/Unknown | 169666 | 40083922 | 5.8 |
| ClassII/Zator | 45 | 2378 | 0 |
| ClassII/Zisupton | 587 | 61622 | 0.01 |
| ClassII/hAT | 25045 | 5821057 | 0.84 |
| Total | 675407 | 364132238 | 52.7 |

## Table S3. Genome Gene Function Annotation

| **Anno_Database** | **Annotated_Number** | **Annotated_Ratio (%)** |
| --- | --- | --- |
| GO_Annotation | 26612 | 84.91 |
| KEGG_Annotation | 24686 | 78.76 |
| KOG_Annotation | 18273 | 58.3 |
| Pfam_Annotation | 27750 | 88.54 |
| Swissprot_Annotation | 26314 | 83.96 |
| TrEMBL_Annotation | 31077 | 99.15 |
| eggNOG_Annotation | 27462 | 87.62 |
| nr_Annotation | 31082 | 99.17 |
| All_Annotated | 31099 | 99.22 |

## Table S4. Genome information for comparative genomic species

| Species | Genome file download address |
| --- | --- |
| Vitis vinifera | http://ftp.ensemblgenomes.org/pub/plants/release-52/fasta/vitis_vinifera/dna/Vitis_vinifera.12X.dna.toplevel.fa.gz |
| Cajanus cajan | https://ftp.ncbi.nlm.nih.gov/genomes/all/GCF/000/340/665/GCF_000340665.1_C.cajan_V1.0/GCF_000340665.1_C.cajan_V1.0_genomic.fna.gz |
| Cicer arietinum | https://ftp.ncbi.nlm.nih.gov/genomes/all/GCF/000/331/145/GCF_000331145.1_ASM33114v1/GCF_000331145.1_ASM33114v1_genomic.fna.gz |
| Populus trichocarpa | https://ftp.ncbi.nlm.nih.gov/genomes/all/GCF/000/002/775/GCF_000002775.4_Pop_tri_v3/GCF_000002775.4_Pop_tri_v3_genomic.fna.gz |
| Ammopiptanthus nanus | ftp://parrot.genomics.cn/gigadb/pub/10.5524/100001_101000/100466/ |
| Medicago truncatula | https://ftp.ncbi.nlm.nih.gov/genomes/all/GCF/003/473/485/GCF_003473485.1_MtrunA17r5.0-ANR/GCF_003473485.1_MtrunA17r5.0-ANR_genomic.fna.gz |
| Glycine soja | https://ftp.ncbi.nlm.nih.gov/genomes/all/GCF/004/193/775/GCF_004193775.1_ASM419377v2/GCF_004193775.1_ASM419377v2_genomic.fna.gz |
| Arachis hypogaea | https://ftp.ncbi.nlm.nih.gov/genomes/all/GCF/003/086/295/GCF_003086295.2_arahy.Tifrunner.gnm1.KYV3/GCF_003086295.2_arahy.Tifrunner.gnm1.KYV3_genomic.fna.gz |
| Dalbergia odorifera | ftp://parrot.genomics.cn/gigadb/pub/10.5524/100001_101000/100760/ |
| Arachis duranensis | https://ftp.ncbi.nlm.nih.gov/genomes/all/GCF/000/817/695/GCF_000817695.2_Aradu1.1/GCF_000817695.2_Aradu1.1_genomic.fna.gz |
| Lupinus angustifolius | https://ftp.ncbi.nlm.nih.gov/genomes/all/GCF/001/865/875/GCF_001865875.1_LupAngTanjil_v1.0/GCF_001865875.1_LupAngTanjil_v1.0_genomic.fna.gz |
| Arabidopsis thaliana | ftp://ftp.gramene.org/pub/gramene/release-65/fasta/arabidopsis_thaliana/dna/Arabidopsis_thaliana.TAIR10.dna.toplevel.fa.gz |
| Spatholobus suberectus | https://ftp.ncbi.nlm.nih.gov/genomes/all/GCA/004/329/165/GCA_004329165.1_ASM432916v1/GCA_004329165.1_ASM432916v1_genomic.fna.gz |
| Arachis ipaensis | https://ftp.ncbi.nlm.nih.gov/genomes/all/GCF/000/816/755/GCF_000816755.2_Araip1.1/GCF_000816755.2_Araip1.1_genomic.fna.gz |
| Glycine max | https://ftp.ncbi.nlm.nih.gov/genomes/all/GCF/000/004/515/GCF_000004515.6_Glycine_max_v4.0/GCF_000004515.6_Glycine_max_v4.0_genomic.fna.gz |

## Table S5. KEGG enrichment analysis of *Dalbergia cultrata* expansion, specific genes, and positively selected genes

See separate Excel file.

## Table S6. Duplication gene’s distribution

| Duplication Types | Number of gene pairs | Number of genes | Percentage of number of gene pairs | Percentage of number of genes |
| --- | --- | --- | --- | --- |
| whole-genome duplication(WGD) | 7670 | 11108 | 29.55% | 40.48% |
| tandem duplication(TD) | 1863 | 3350 | 7.18% | 12.21% |
| proximal duplication (PD) | 901 | 1893 | 3.47% | 6.90% |
| transposed duplication (TRD) | 5813 | 5524 | 22.40% | 20.13% |
| dispersed duplication (DSD) | 9706 | 5568 | 37.40% | 20.29% |

## Table S7. 9 Cluster GO or KEGG enrichment results

See separate Excel file.

## Table S8. Resequencing information

| Latin name | elevation (m) | latitude (N) | longitude (E) | origin | ReadSum | Mapped ReadSum | Map Rate (%) | GC (%) | Q30 (%) | source ID |
| --- | --- | --- | --- | --- | --- | --- | --- | --- | --- | --- |
| *Dalbergia sissoo* | 1176 | 25.40 | 101.52 | Yuanmou, Yunnan province, China | 895,858,393 | 533,711,303 | 59.58 | 35.34 | 95.29 | YDHT |
| *Dalbergia cultrata* | 934 | 22.61 | 100.64 | Puer district, Yunnan province, China | 520,743,140 | 515,391,259 | 98.97 | 34.68 | 95.43 | SM5 |
| *Dalbergia cultrata* | 934 | 22.61 | 100.64 | Puer district, Yunnan province, China | 504,191,159 | 494,659,525 | 98.11 | 35.01 | 94.32 | SM4 |
| *Dalbergia cultrata* | 983 | 22.61 | 100.66 | Puer district, Yunnan province, China | 529,191,779 | 517,408,002 | 97.77 | 34.75 | 95.36 | SM3 |
| *Dalbergia cultrata* | 983 | 22.61 | 100.66 | Puer district, Yunnan province, China | 520,706,442 | 509,594,601 | 97.87 | 34.82 | 95.71 | SM2 |
| *Dalbergia cultrata* | 983 | 22.61 | 100.66 | Puer district, Yunnan province, China | 483,286,603 | 473,139,845 | 97.9 | 34.87 | 95.57 | SM1 |
| *Dalbergia cochinchinensis* | 85 | 18.70 | 108.80 | Jianfengling district, Hainan province, China | 865,188,116 | 499,492,221 | 57.73 | 35.82 | 95.47 | JZHT |
| *Dalbergia odorifera* | 85 | 18.70 | 108.80 | Jianfengling district, Hainan province, China | 849,168,212 | 579,757,834 | 68.27 | 35.09 | 95.28 | JXHT |
| *Dalbergia hupeana* | 581 | 31.86 | 104.74 | Jiulianshan district, Jiangxi province, China | 818,187,326 | 479,070,421 | 58.55 | 35.01 | 95.8 | HT |
| *Dalbergia dongnaiensis* | 240 | 14.50 | 102.15 | Khonburi district, Nakhonrachassima province,Thailand | 144,276,469 | 69,427,740 | 48.12 | 35.25 | 85.66 | ERR7625924 |
| *Dalbergia cochinchinensis* | 240 | 14.91 | 102.15 | Khonburi district, Nakhonrachassima province,Thailand | 81,209,599 | 44,429,415 | 54.71 | 36 | 83.53 | ERR7584956 |
| *Dalbergia lanceolaria* | 365 | 17.90 | 98.78 | Li district, Lamphun province,Thailand | 135,615,161 | 53,643,903 | 39.56 | 34.48 | 88.23 | ERR7574536 |
| *Dalbergia oliveri* | 359 | 18.93 | 99.05 | Sansai district, Chiangmai province,Thailand | 79,530,645 | 45,515,926 | 57.23 | 34.84 | 83.63 | ERR7571223 |
| *Dalbergia oliveri* | 367 | 14.95 | 98.78 | Saiyoke district, Kanchanaburi province,Thailand | 76,287,202 | 43,798,898 | 57.41 | 34.62 | 83.47 | ERR7570999 |
| *Dalbergia cultrata* | 230 | 14.88 | 98.82 | Thongphaphum district, Kanchanaburi province,Thailand | 59,628,702 | 56,713,069 | 95.11 | 34.27 | 86.68 | ERR7570470 |
| *Dalbergia cultrata* | 180 | 16.52 | 99.24 | Kosampi district, Kamphaengphet province,Thailand | 119,328,654 | 112,054,308 | 93.9 | 33.77 | 81.65 | ERR7565649 |
| *Dalbergia nigrescens* | 8 | 13.77 | 100.31 | Phutthamonthon district, Nakhonpathom province,Thailand | 109,399,561 | 60,166,319 | 55 | 35.23 | 91.8 | ERR7565127 |
| *Dalbergia oliveri* | 382 | 18.94 | 99.05 | Sansai district, Chiangmai province,Thailand | 107,300,737 | 63,486,109 | 59.17 | 34.37 | 92.29 | ERR7565126 |
| *Dalbergia cana* | 382 | 18.94 | 99.05 | Sansai district, Chiangmai province,Thailand | 108,296,561 | 59,798,966 | 55.22 | 34.28 | 92.33 | ERR7565123 |

## Table S9. Primer sequences used in RT-qPCR experiments

| GeneName | Left Primer | Right Primer | ProductSize |  |
| --- | --- | --- | --- | --- |
| Dcu08G000830 | CAACACCATGCCTTTGTCGA | GCATGAGAAGGGAAAGAGCG | 92 |  |
| Dcu06G028310 | ACCACAACAAGAGCAGCAAG | ACGCAACGCCTCAATTTCAT | 104 |  |
| Dcu01G040030 | GTTTGAGCAGAGCGGAGATG | ACGGTCAACTGGTTTTCTGC | 127 |  |
| Dcu07G024340 | GGCACCCTGTTCCTCTGTAT | CCTCAAGGCGCATGAATCTC | 82 |  |
| Dcu04G001800 | TGGATCAGACAGCCTTGGTT | CCACTTAAATGCATGCCGGT | 90 |  |
| Dcu01G003570 | TTGGGCCTAGAAAACAACGG | AGCCTCGTCCTCATCATTGT | 87 |  |
| Dcu01G033940 | CTCCTCTGTGACTCCTGCAA | GTGAACAGGGGAGAGGGAAA | 117 |  |
| Dcu10G014970 | GGTTGTTCCGTGGTCCTCTA | AATCGTGATCCTCGTCGTGA | 149 |  |
| Dcu09G016550 | GTCGCACAACTGGTATCGTC | TCGAAGGATGGCATGTGGTA | 95 | ACT |
| Dcu10G018280 | GTACCTGCCTTTGGATCTGC | CGCTCTGTCTTCAAGGATGC | 87 | 60SrRNA |
| Dcu09G001470 | GGTTTGGCATTGTTGAGGGT | GTGCTGCTGGGAATGATGTT | 133 | GAPDH |
